# Gene signatures associated with exosomes as diagnostic markers of postpartum depression and their role in immune infiltration

**DOI:** 10.3389/fendo.2025.1542327

**Published:** 2025-07-17

**Authors:** Jianbo He, Hong Chen, Kaiming Duan, Sikandaier Wushouer, Xiaowei Wang, Yaxuan Li, Xingang Qin

**Affiliations:** ^1^ Department of Anesthesiology, The Fifth Affiliated Hospital of Xinjiang Medical University, Urumqi, China; ^2^ Department of Anesthesiology, Third Xiangya Hospital of Central South University, Changsha, China

**Keywords:** exosomes, bioinformatics analysis, immune infiltration, diagnostic model, biomarkers

## Abstract

**Background:**

Postpartum depression (PPD) is a significant mental health challenge for new mothers, with diverse and unclear causes. Exosomes significantly contribute to the pathogenesis, identification, treatment outcome determination, and intervention of PPD. However, the functions of exosome-related genes (ERGs) in PPD remain to be fully elucidated. This study examines the potential impact of ERGs on PPD and develops a set of diagnostic tools based on them.

**Methods:**

We acquired and prepared several gene expression datasets from the Gene Expression Omnibus (GEO). Our analysis focused on genes that closely interact with the extracellular matrix. Using advanced techniques, including the *limma* package, we identified differential expression and conducted enrichment analyses of Gene Ontology (GO) and Kyoto Encyclopedia of Genes and Genomes (KEGG). Furthermore, we employed logistic regression, random forest (RF) classifiers, and least absolute shrinkage and selection operator (LASSO) regression to screen critical genes.

**Results:**

We identified 44 exosome-related differentially expressed genes (ERDEGs) that play key roles in synaptic signal transmission, hormone fluctuations, and inflammatory responses. Ten genes, including *TPP2*, *AKR1B1*, *CD59*, *PARK7*, *PLXNB2*, *HLA-B*, *FAH*, *NDST1*, *SCARB1*, and *HNRNPA2B1*, were established using logistic regression analysis, RF method, and LASSO regression. In these two sets of data, the manifestations of *PARK7* and *HNRNPA2B1* differed. The analysis showed that the significant enrichment of gene sets was strongly associated with high-risk scores, particularly in the metabolic (phospholipid metabolism) and neural (mitochondrial translation) pathways. Gene set variation analysis (GSVA) revealed four prominent pathways: MYC targets V2, pancreatic beta cells, unfolded protein response, and oxidative phosphorylation. Single-sample gene set enrichment analysis (GSEA) showed that immune cells demonstrated different degrees of infiltration among at-risk and low-probability risk subsets of immature B cells, regulatory T cells), and T follicular helper cells.

**Conclusions:**

ERDEGs significantly contribute to PPD occurrence. Our diagnostic model demonstrated high accuracy and potential for use in medical practice. Future research with larger samples is warranted to validate these conclusions and identify effective targets that may affect these pathways during treatment to improve the therapeutic effect.

## 1 Introduction

Postpartum depression (PPD) is a psychological disorder that affects many women after childbirth. This condition is characterized by intense depressive episodes that emerge within a month of delivery and can persist for years without proper intervention (1). This condition affects the mother’s well-being and adversely affects the infant’s development and the overall family dynamics (2). PPD affects approximately 17.7% of the global population and 21% of our nation’s population (3, 4). Despite the prevalence of PPD, the rate of diagnosis and cure has not advanced. The key reason for this is the inadequate clear biomarkers, which has led to the innovation of diagnostic methods and treatment plans (5).

Exosomes are small extracellular vesicles secreted by various cell types that have emerged as biomarkers and therapeutic targets, showing great potential in numerous diseases such as cancer and neurodegenerative and cardiovascular diseases (6). These vesicular structures host a diverse array of proteins, lipids, and nucleic acids that mirror the physiological state of their mother cells. Recent research has highlighted the important role of exosome-related genes (ERGs) in manipulating immune responses, cellular interactions, and metabolic pathways, making them attractive targets for intervention to elucidate the mechanisms of various complex diseases, such as PPD. ERGs can directly or indirectly affect CD4 + and CD8 + T cells, thus stimulating or inhibiting the proliferation and biological activity of these immune cells (7).

Under the conditions of PPD, studying the ERG hypothesis may reveal unknown molecular pathways, which may point to the early discovery of biomarkers for tailoring medical protocols. Exosomal microRNAs (miRNAs) and proteins are involved in the pathophysiological processes of major depressive disorder (MDD) and other psychiatric disorders, suggesting that they may have similar functions in PPD (8). For example, exosomal miR-139-5p has been implicated in the regulation of neuroinflammation in MDD, highlighting the relevance of exosomal components in mood disorders (9).

We chose PPD as a disease model because of its high incidence, profound effects on maternal and infant health, and the current lack of objective molecular diagnostics. In addition, the etiology and pathogenesis of PPD have not been fully elucidated, and new biomarkers and therapeutic targets are urgently needed. Recent evidence suggests that exosomes and their related genes play a key role in neuropsychiatric disorders, but their function in PPD has not been fully explored. Therefore, focusing on PPD can help fill important clinical gaps and open up the possibility of new diagnostic and therapeutic approaches based on exosome biology.

Although exosomes-associated genes (ERGs) have been shown to play a role in immune regulation and neuropsychiatric disorders such as major depressive disorder (MDD), the relevant studies focusing on postpartum depression (PPD) are extremely limited. To date, there are few systematic reports on the specific molecular mechanisms and extracellular vesicle-derived biomarkers of PPD, and only sporadic studies have provided indirect evidence. This significant research gap highlights the need for systematic screening and identification of PPD-specific exosome gene signatures. This study aimed to examine the potential effects of ERGs on PPD and to construct diagnostic systems based on them.

## 2 Materials and methods

### 2.1 Data download and preprocessing

Using the GeoQuery toolkit in R, we obtained the gene expression dataset of patients with PPD (10) using the R package GEOquery (11) When studying the GSE45603 dataset, we specifically selected the patient population that exhibited PPD traits and compared them with individuals with normal performance on the normal postpartum mood index. In total, 43 data samples were collected: 16 and 27 from women in the PPD and normal postpartum mood categories, respectively.

The data platform for GSE45603 was the GPL10558Illumina HumanHT-12 V4.0 expression beadchip. A microarray GPL platform file was used to annotate probe names in the dataset (**Supplementary Table S1**).

GeneCards (12) database (https://www.genecards.org/) presents detailed data on human genetic codes. In order to obtain exosome-related genes, the search term “exosomes” was used to search from genecards, we selected exosomes as the query code and further screened out ERGs that only encoded proteins and had a correlation score exceeding 2. Cumulatively, 778 ERGS of energy units were obtained.

Overall, 121 ERGs were retrieved from the published literature (13) We obtained 880 ERGs by combining all the ERGs obtained and removing duplications; the detailed data are presented in **Supplementary Table S1**.

### 2.2 Differential expression analysis

To discern potential mechanisms and associated biological characteristics and routes of genes participating in PPD, we first used the *limma* package (14) to perform differential analysis on the postpartum depression dataset GSE45603 to obtain Differentially expressed genes (DEGs) between different groups (PD/Control) of the postpartum depression dataset. A threshold of |logFC| > 1 was used in the preliminary analysis, but at the stage of identifying ARDEGs, the choice of threshold for fold change in the differential analysis was appropriately relaxed in order to include as many differentially expressed genes as possible. Finally, the genes with |logFC| > 0 and p value < 0.05 were selected as the differentially expressed genes (DEGs) for further study. genes with logFC > 0 and p value < 0.05 were up regulated genes. genes with logFC < 0 and p value < 0.05 were down regulated genes.

To screen out DEGs with | log FC | > 0 and p < 0.05, the GSE45603 PPD dataset was processed using ERGS; ERDEGs and Wayne diagrams were produced by comparative analysis. These analysis results are presented in the form of a volcano map using the ggplot2 package of R language and in the form of a heat map using the pheatmap package.

### 2.3 Functional enrichment analysis using gene ontology and pathway enrichment analysis using the Kyoto Encyclopedia of Genes and Genomes (KEGG)

GO (15) analysis is frequently used for performing extensive functional enrichment research on biological process (BP), molecular functions (MF), and cellular component (CC). KEGG (16) is widely used to store genomes, biological pathways, diseases, and drug information databases. ERDEGs were subjected to GO annotation and KEGG pathway enrichment analysis using the R package clusterProfile (17). The criteria for initial screening were set as *p* < 0.05 and a false discovery rate value (q.value) < 0.25 was to be considered significant.

### 2.4 Screening of key genes

Initial analysis of the ERDEGs was conducted using univariate logistic regression to identify the important genes and establish the prediction model. The results showed that the p-value was < 0.05, which was used as the benchmark for screening. The ERDEGs that met the criteria were included in the subsequent random forest analysis. The integrated decision tree classifier (18) is a method that combines several PPD decision trees based on the principle of ensemble learning. This method belongs to the bagging technology category within the framework of ensemble learning and involves the combination of various algorithms. Random forest is another common modeling technique. A series of decision trees are used to form a decision forest. When predicting specific data points, the prediction data provided by each tree are summarized, and the final prediction value is extracted from many predictions through a voting mechanism. After screening, the ERDEG expression levels in the dataset GSE45603 were identified using univariate logistic regression. Subsequently, using the random forest toolkit, a model was built (19), with the parameters set.seed (234) and ntree = 500. The median decrease in the Gini coefficient represents an average reduction. The purity of a node was measured using the Gini index. When the value of the Gini coefficient increases, the purity decreases accordingly, which means that the proportion of harmful components increases. Meandecreasegini represents the average reduction in the impurities of the variable separating the nodes for all trees, and a larger Meandecreasegini represents more important variables for our grouping. We then cross-validated the data 10 times by performing five iterations and integrating the cross-validation curves to determine the appropriate number of variables. The function of the cross-validation method is to use different training sets or validation set partitions to perform multiple groups of different training or validation sets on the model to address the problem of the individual test results being too one-sided and the training data being insufficient. We used our training set to perform cross-validation to screen out the relevant variables with low error and employed the Meandecreasegini criterion to select the key variables for further study.

Consequently, we used the R package glmnet (20) to perform LASSO (Least absolute shrinkage and selection operator) (21) regression analysis based on random forest screening with set.seed (500) as the parameter. To avoid overfitting, the loop number was set to 200. By introducing a penalty factor (the multiplication of lambda and the magnitude of the slope) into the traditional linear regression framework, LASSO regression analysis effectively alleviates the overfitting problem of the model, thereby enhancing its universal adaptability. The effectiveness of LASSO regression analysis was demonstrated through visualization using diagnostical model layouts and variable track diagrams. ERDEG was the pivot for subsequent analysis in the final draft of the LASSO regression model.

### 2.5 Key genes to construct the diagnostic logistic regression model

A logistic discriminant analysis model is often used to explore the correlation between independent and dependent variables when the dependent variable exhibits two states. We collected all the important genes and built a logistic regression model with multiple variables to calculate the weight of each important gene by multiplying the expression level of the gene by a specific coefficient. After adding these products, we obtained a risk profile score for each sample. According to the median value of the RiskScore, the disease classifications within the dataset were categorized into two risk strata: high and low. Patients in the dataset were segregated into elevated- and reduced-risk categories. The RiskScore was calculated using the following formula:


$$\text{RiskScore }=\;\sum_{\text{i}}\text{Coefficient }\text{gen}{\text{e}}_{\text{i}}*\text{mRNAExpression }\text{gen}{\text{e}}_{\text{i}}$$

A nomogram (22) A calibration curve was constructed through calibration analysis to assess the precision and discriminability of the model derived from the outcomes of multivariate logistic regression.

Decision curve analysis (DCA) is a straightforward technique used to assess the performance of clinical forecasting models, diagnostic examinations, and molecular biomarkers. The R package ggDCA was used to generate DCA (23)

The receiver operating characteristic (ROC) (24) serves as a visual analytical instrument that can identify the optimal model, reject the runner-up model, or determine the most suitable cutoff within the same model. The ROC curve is a comprehensive indicator of the relationship between two continuous variables of sensitivity and specificity, and the interaction between both is revealed by the combined method. The area under the curve (AUC) is typically between 0.5 and 1. Finally, using the R language package pROC, a logistic discriminant analysis was fitted to the dataset GSE45603, and the corresponding ROC curve was plotted to estimate the AUC to explore the efficacy of the logistic risk score in the risk assessment of PPD diagnosis. In statistics, an AUC value close to 1 indicates a superior diagnostic efficacy. AUC scores ranging from 0.5 to 0.7 suggest low accuracy, whereas those falling between 0.7 and 0.9 denote moderate accuracy. An AUC exceeding 0.9 indicates high accuracy.

### 2.6 Validation of gene expression disparities and analysis of functional homology among pivotal genes

The Mann–Whitney U test, alternatively referred to as the Wilcoxon rank-sum test, was employed to investigate substantial variations in the expression levels of key genes between each group (PPD vs. control) within the GSE45603 dataset. Using the ggplot2 toolkit of R, an intergroup comparison chart was then constructed to present the final effect of the difference test.

Furthermore, the Spearman method was used to explore the correlation between the expression of important genes in the GSE45603 dataset. Visual analysis was performed using the PheatMap package in the R software. Correlation coefficients with an absolute value < 0.3 imply a weak or negligible relationship. Those ranging from 0.3 to 0.5 indicate a faint correlation. Values between 0.5 and 0.8 suggest a moderate degree of correlation, and coefficients exceeding 0.8 denote a robust correlation.

The semantic comparison method provided by GO annotation, a means of evaluating the similarity between genes and genomes, occupies a central position in many bioinformatics studies, and its analysis technology is widely used. The GOSemSim software suite was used to estimate the GO semantic similarity of important genes (25) The ggplot toolkit was used to graphically display the results of the functional correlation studies, aiming to uncover the correlation between functionally linked genes. Finally, the software package R-Circos (26)

### 2.7 Gene set enrichment analysis

GSEA (27) The first step was to arrange the logFC values of the genes in descending order. Subsequently, we assessed the clustering of all genes associated with the logFC values using the ClusterProfiler tool. In the GSEA, the key parameter values included seed setting based on 2022, 5000 times of calculations to ensure that each gene set contained at least 10 genes, and the upper limit of the number of genes in the gene set was set to 500. The BH method was adopted for correction. We derived from the Molecular Signatures Database (MSigDB) (28) The criterion for screening out a significant concentration was based on an adjusted p-value of < 0.05 and a false discovery rate values (q-value) of < 0.25. The BH method was the adopted p-value adjustment strategy.

### 2.8 Gene set variation analysis

GSVA (29) We used various methods to investigate whether a concentration phenomenon existed in different samples. We successfully obtained the gene combination “h.all.v7.4. symbols.gmt.” Based on the in-depth mining of GMT information in the MSigDB database, we performed a comprehensive GSVA on each subgroup in Dataset A, aiming to reveal the differentiated performance of their functions. The screening criteria showed clear enrichment p-value < 0.05.

### 2.9 Immune infiltration analysis

We employed the single-sample GSEA (ssGSEA) method (30) to measure the comparative prevalence of various immune-cell infiltrates. We identified and labeled various types of permeable immune cells, such as T cells with CD8 + markers, dendritic cells, macrophages, regulatory T cells (Tregs), and other subtypes of human immune cells. The enrichment index calculated using ssGSEA revealed a comparison of the infiltration levels of various immune cells in each sample. The ssGSEA method implemented in the GSVA toolkit of the R language was used to quantitatively describe the content of various types of immune cells present within individual samples. Box plots revealed the difference in the infiltrating abundance of 28 immune cells between the high- and low-risk logistic regression score groups in the GSE45603 dataset. Furthermore, we analyzed the gene expression profiles in the GSE45603 dataset, explored the association between immune cells with significantly different risk scores and key genes in the logistic regression model, and constructed a scatter plot using the ggplot2 package of R to reveal this association.

### 2.10 Regulatory network analysis of key genes

The public comparative toxicogenomics database (CTD) (31) To enhance the comprehension of health-related human data, we used the CTD to forecast possible pharmaceuticals or small molecular substances that could engage with pivotal genes, applying a “Reference Count” greater than 2 as the filter criterion for identifying messenger RNA (mRNA)-drug interaction pairs. The Cytoscape tool was used to depict the mRNA-drug interaction network.

ENCORI database (32) the relationship between RNA binding proteins (RBP)-non-coding (ncRNA) and RBP-mRNA was inferred from CLIP-seq and decompotome sequencing (for plants) data extraction, providing various visualization tools for examining miRNA targets. The ENCORI database was employed to forecast the miRNAs that engage with essential genes, and the mRNA-miRNA interaction pairs were filtered based on the criterion of pancancerNum exceeding 14. Cytoscape was used to depict the mRNA-miRNA interaction network.

Additionally, we used the EnCORI database to predict RBPs that interact with important genes and used ClusterNum > 24 as a screening criterion to select mRNA-RBP interaction pairs. The Cytoscape tool was used to depict the network architecture of the mRNA-RBP interactions.

Using the CHIPBase database (version 3.0) (33) (https://rna.sysu.edu.cn/chipbase/), after ChIP - seq analysis of DNA-binding proteins, we detected thousands of base-binding motifs along with their respective interaction locations, revealing the regulatory links between millions of transcription factors (TFs) and genes. As a criterion for screening mRNA-TF interaction pairs, the count of TF binding to the key gene observed was required to be zero. If the total number of samples collected in the upstream and downstream of the gene exceeds ten, this condition is regarded as a necessary element in the screening. Cytoscape was used to visualize the interaction network between mRNA and TFs.

### 2.11 Statistical analysis

All data manipulations and evaluations were conducted using the R programming language (version 4.2.2). Continuous data were presented as means and standard deviations. The Wilcoxon signed-rank test was used to compare the two groups. The Kruskal–Wallis test is suitable for the controlled analysis of three or more groups. Spearman rank correlation coefficients were used for data analysis unless otherwise specified. The p-values of all statistical analyses were bidirectional, and p-values < 0.05 were considered statistically significant.

## 3 Results

### 3.1 Technology roadmap

**Figure 1:**
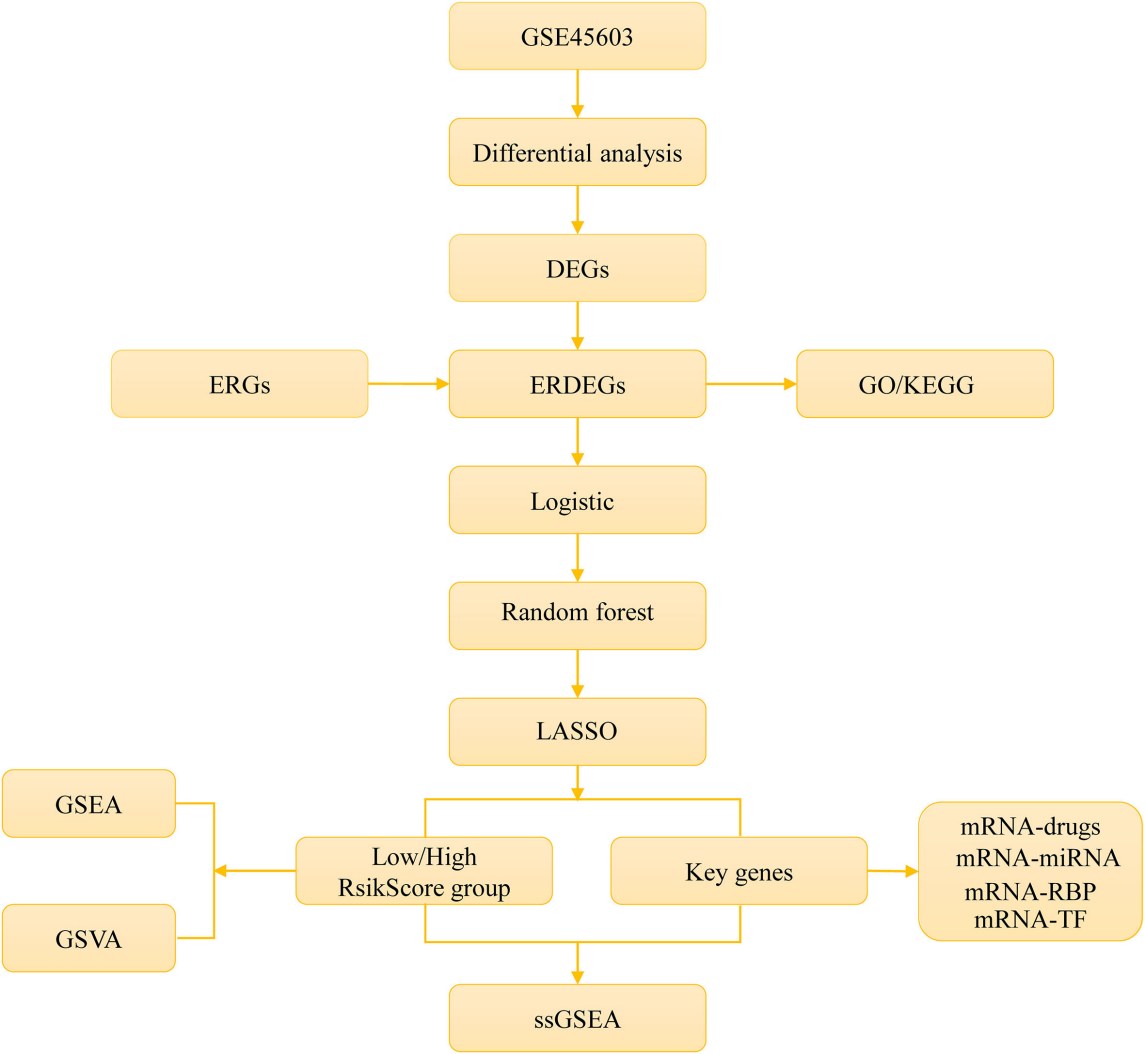
Technology roadmap.

The overall workflow of this study is illustrated in **Figure 1**.

### 3.2 Data preprocessing

**Figure 2:**
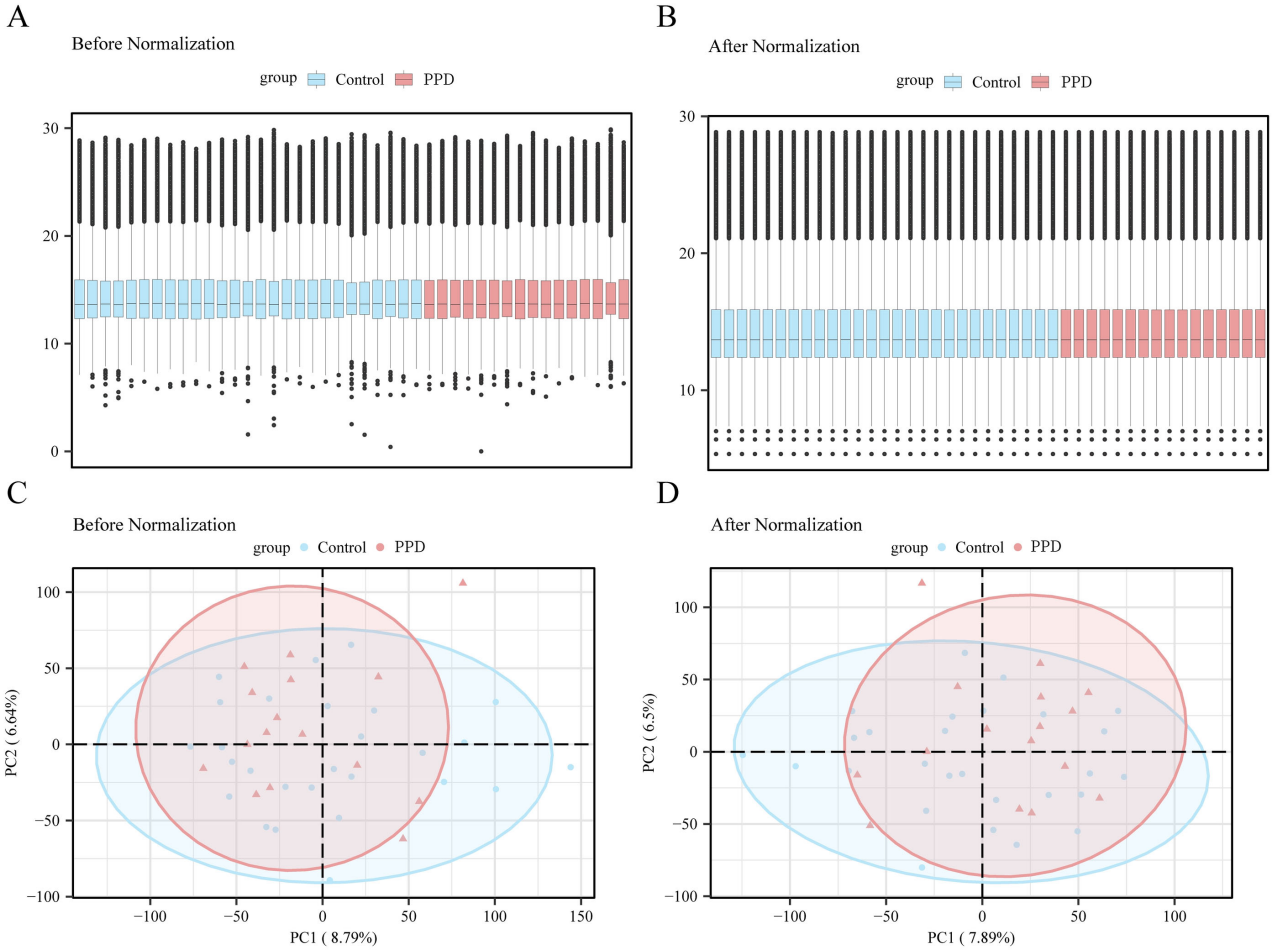
Data preprocessing. **(A)** Boxplot of gene expression distribution for GSE45603 dataset before batch effect correction. **(B)** Boxplot of improved inter-sample agreement after correction. **(C)** principal component analysis (PCA) plot of GSE45603 dataset before correction to reflect sample variation due to batch effect. **(D)** The corrected principal component analysis chart shows that the batture-related variation is effectively reduced, and the postpartum depression (PPD) group is more clearly distinguished from the control group. Light blue represents the control group and light red represents the PPD group. PPD, postpartum depression; PCA, principal component analysis; GSE45603, a gene-expression data set from the Gene Expression Omnibus.

In the first step, the Surrogate Variable Analysis toolkit of the R language was used to perform variable selection on the GSE45603 dataset, and the filtered GSE45603 was generated. Using box plots and principal component analysis charts, the datasets from the pre- and post-stage screening were then compared and analyzed (**Figures 2A–D**). Box plot and principal component analysis showed that batch-related effects among the samples in the GSE45603 dataset were largely resolved after excluding the batch factor.

### 3.3 Differential expression analysis of genes related to external body

The *limma* toolkit was used to conduct an in-depth comparative analysis of the information in the GSE45603 dataset to investigate the variations in gene transcription levels between different categories (PPD/Control) in the PPD dataset. The aim is to identify genes with altered expression (DEGs) among the distinct classifications (PPD/Control). Dataset GSE45603 comprises 11,819 genes, with 966 exhibiting differential expression based on the criteria of | logFC | > 0 and p-value < 0.05. Within this subset, 578 genes were upregulated in the PPD group (the control group showed reduced expression with positive logFC values), and 388 genes were downregulated in the PPD group (the control group showed increased expression with negative logFC values).

**Figure 3:**
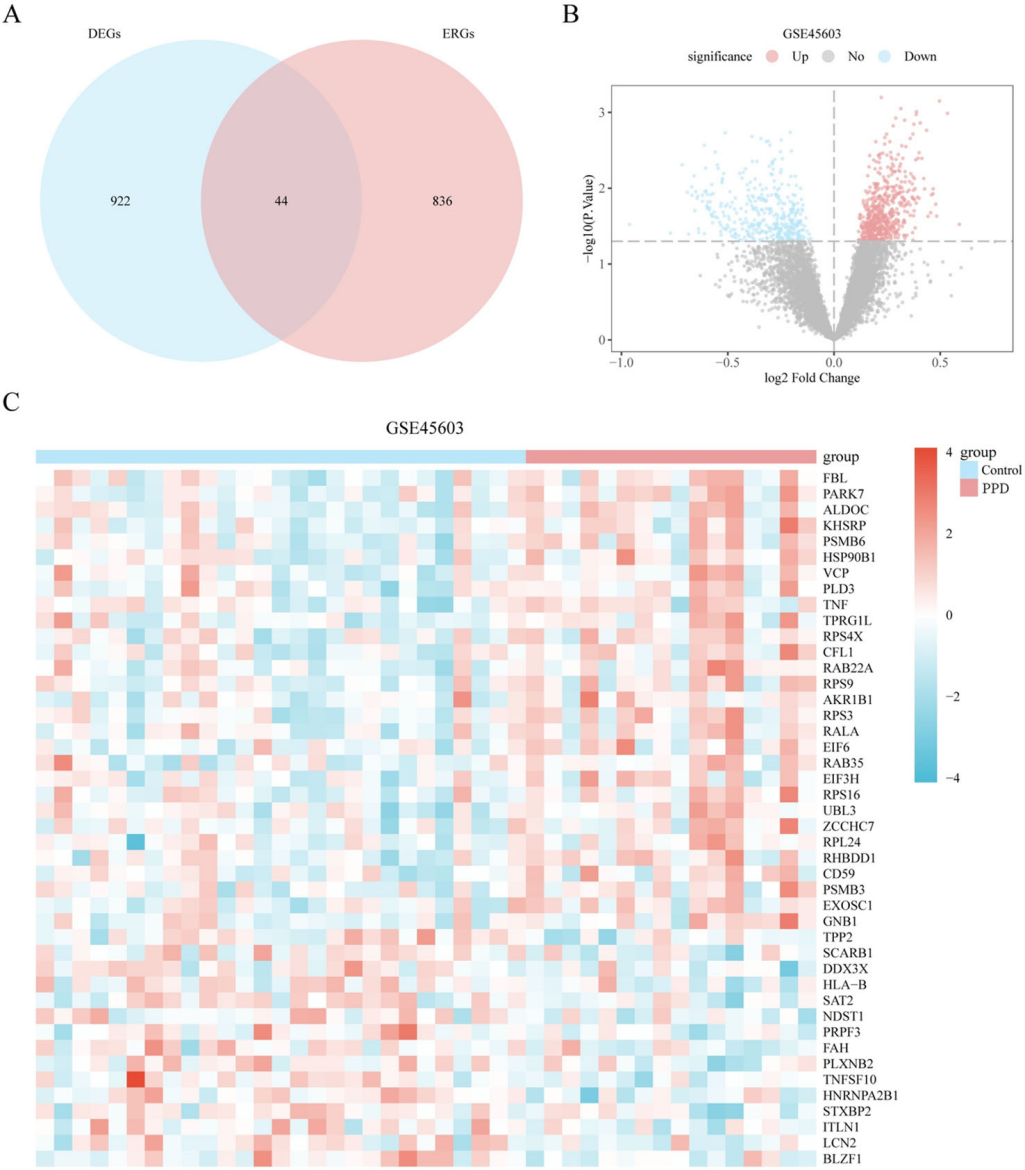
Differential expression analysis of genes related to the external body. **(A)** Venn diagram of differentially expressed genes (DEGs) and exosome-related genes (ERGs) in dataset GSE45603. **(B)** Volcano plot of differentially expressed genes analysis between different groups (postpartum (PPD)/Control) of dataset GSE45603. **(C)** Expression heatmap of exosome related differentially expressed genes (ERDEGs) between different groups (PPD/Control) of dataset GSE45603. Light red represents the PPD group, and light blue represents the control group. Red represents high expression, and blue represents low expression, respectively, on the heat map. PPD, postpartum depression; DEGs, Differentially expressed genes; ERGs, Exosome-related genes; ERDEGs, Exosome related differentially expressed genes.

To identify ERDEGs, we filtered dataset GSE45603 for DEGs with | logFC | > 0 and a p-value < 0.05 and intersected these with extracellular vesicle-related ERGs to yield 44 ERDEGs (**Supplementary Table S2**). A Venn diagram illustrating this process is shown in **Figure 3A**. Difference analysis results for dataset GSE45603 are displayed in a volcano plot (**Figure 3B**). The expression differences of 44 ERDEGs genes in each category (PPD/Control) of dataset GSE45603 were explored through the information revealed by the Venn map, and the corresponding heat map was drawn using the PheatMap package of the R language, thus visually displaying the detailed information of these differential expressions (**Figure 3C**).

### 3.4 Functional enrichment analysis (GO) and pathway enrichment analysis (KEGG)

**Figure 4:**
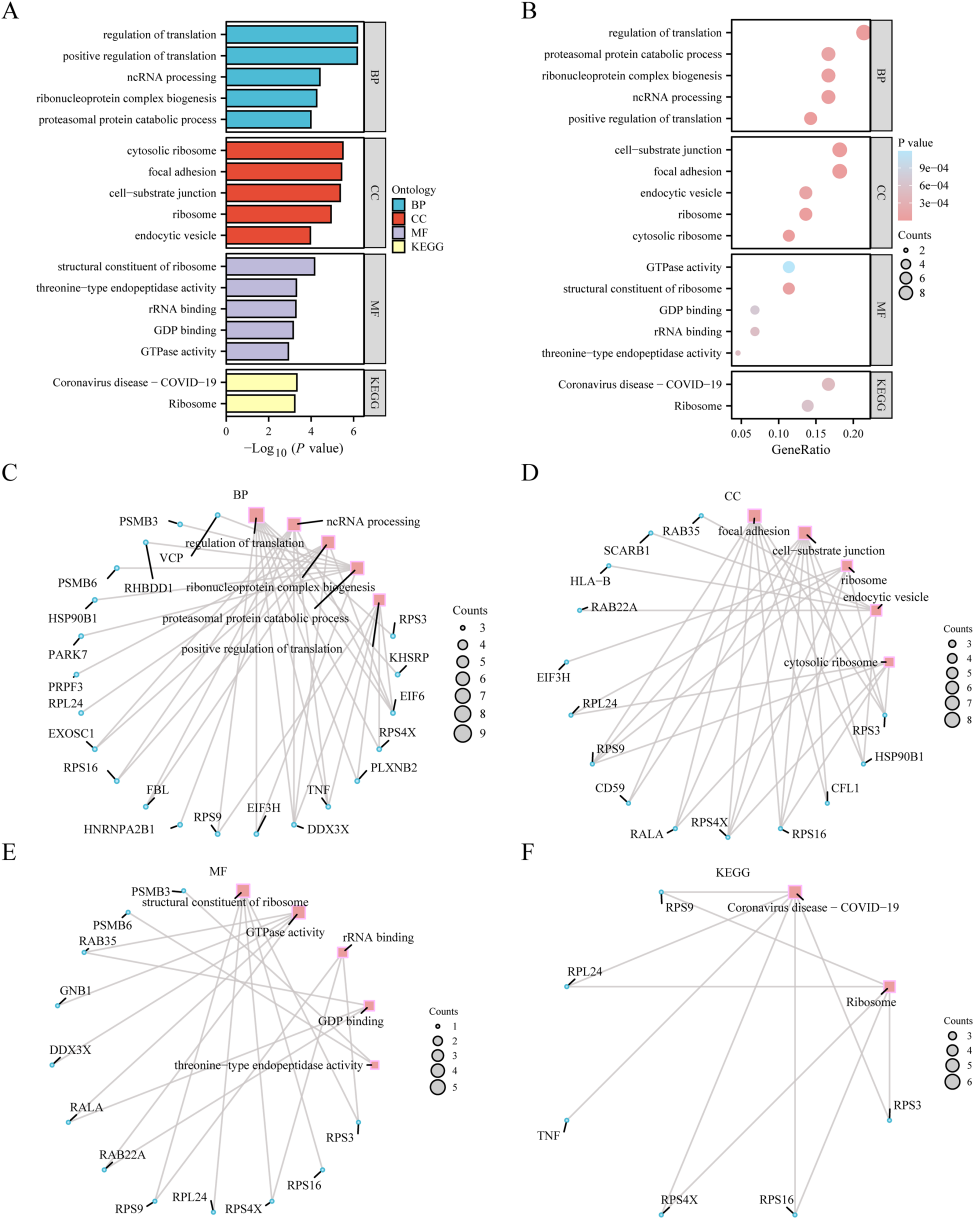
GO enrichment analysis and KEGG enrichment analysis. **(A)** Bar graph of gene ontology (GO) enrichment analysis results of exosome-related differentially expressed genes (ERDEGs). **(B)**. Bubble plot of GO enrichment analysis results of ERDEGs. The ordinate is GO terms. **(C–E)** Network diagram of GO enrichment analysis results of ERDEGs (**C** BP, **D**: CC, **E**: MF). In the network diagram **(C–E)**, red dots represent specific pathways, and blue dots represent specific genes. **(F)** Network diagram of KEGG enrichment analysis results of ERDEGs. The screening criteria for GO enrichment items were p value< 0.05 and false discovery rate value (q.value) < 0.25, and the Benjamini-Hochberg (BH) method was used for p-value correction. GO, Gene Ontology; BP, Biological process; CC, Cellular component; MF, Molecular function; KEGG, Kyoto encyclopedia of genes and genomes; ERDEGs, Exosome-related differentially expressed genes; BH, Benjamini-Hochberg.

After the GO richness analysis, we explored the correlation between the 44 ERDEGs and A considering BP, CC, MF, and specific biological pathways. Forty-four ERDEG indicators were used for GO enrichment analysis, and the results are presented in **Supplementary Table S2** (GO section). The 44 ERDEGs were mainly involved in translation control, processing of non-coding RNA, formation of ribonucleoprotein complexes, proteasome-mediated protein degradation, and positive regulation of translation. They play a key role in cellular processes, such as focal adhesion, cell-matrix interactions, ribosome activity, vesicles formed by endocytosis, and ribosomes in the cytoplasm and exhibit other functional features, such as ribosome composition, GTPase activity, binding to rRNA, binding to GDP, and threonine-type endopeptidase activity. KEGG enrichment analysis of 44 ERDEGs revealed the remarkable richness of these ERDEGs in the coronavirus disease-COVID-19 and ribosomal KEGG pathways. Using a graphical format, we demonstrated the effectiveness of GO functional enrichment analysis (**Figure 4A**) and bubble diagrams (**Figure 4B**). We constructed a BP network diagram (**Figure 4C**), CC (**Figures 4D, E**), and KEGG (**Figure 4F**). The functional network of GO genes was drawn according to enrichment analysis. These tracks indicated the relevant molecules and their corresponding annotation entries. With the expansion of the node size, the number of molecules involved in the entries under it increases accordingly.

### 3.5 Screening of key genes

**Figure 5:**
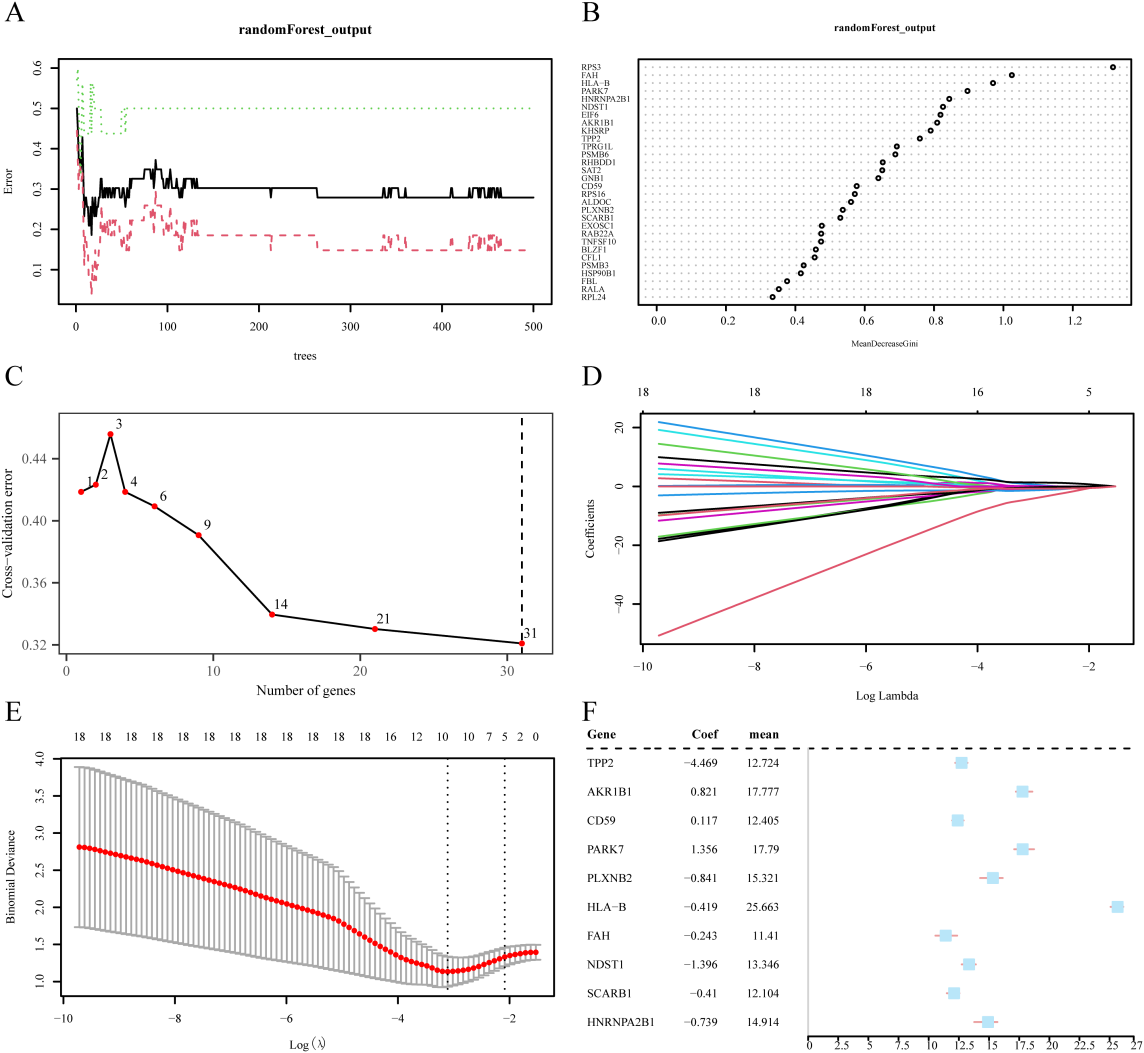
Screening of key genes. **(A)** Plot of model training error of random forest algorithm. **(B)** MeanDecreaseGini scatter plot of ERDEGs (in descending MeanDecreaseGini order). **(C)** Cross-validation error plot. **(D)** Diagnostic model plot of Least absolute shrinkage and selection operator (LASSO) regression model. **(E)** Variable trajectory plot of LASSO regression model. **(F)** Forest plot of key genes in LASSO regression model. ERDEGs, Exosome-related differentially expressed genes; LASSO, Least absolute shrinkage and selection operator.

To refine the selection of pivotal genes, we initially applied logistic regression analysis, which identified 44 ERDEGs (p < 0.05), leading to the final retention of 31 ERDEGs (**Supplementary Table S3**). Subsequently, we used the random forest algorithm to analyze the expression of 31 ERDEGs in the PPD/control group in the GSE45603 dataset. The initial sample size was set to 234, and the number of decision algorithm models was set to 500. The error rates of the algorithm models were plotted over time (**Figure 5A**). The study revealed that the observed error reached its lowest point and stabilized when the number of decision trees reached approximately 300. Subsequently, we plotted a graph that measured the Gini index of reduction. During screening for important genes, 31 ERDEG were identified (**Figure 5B**). The median reduction in the Gini coefficient is referred to as the MeandecreaseGini coefficient. The purity of a node was measured using the Gini index. The degree of the Gini coefficient increases, and the degree of purity is correspondingly reduced, suggesting that more mixed components are present. Meandecreasegini represents the average reduction in impurities in the variable separation nodes of all trees. Larger mean and decreased Gini values indicate that the genes are critical for our grouping (PPD/Control), suggesting their significant impact on the diagnostic accuracy of GSE45603. Subsequently, we adopted a five-time ten-fold cross strategy for validation and plotted the validation error to determine the optimal number of genes (**Figure 5C**). After image analysis, the error curve reached its lowest point when the gene count was 31. Based on this, the Meandecreasegini index was integrated to select key genes and conduct in-depth research. The final results showed that after intelligent algorithm screening, 31 ERDEG factors with a significant impact on the diagnosis of PPD were identified (**Figures 5B, C**). The factors were rearranged according to their magnitude of influence as follows: rps3, *FAH, HLA-B, PARK7, HNRNPA2B1, NDST1, EIF6, AKR1B1, KHSRP, TPP2, TPRG1L, PSMB6, RHBDD1, SAT2, GNB1, CD59, RPS16, PLXNB2, SCARB1, EXOSC1, RAB22A, TNFSF10, CFL1, FBl, RALA, RPL24, and RPS4X*. This ranking reflects the relative importance of each factor during the diagnosis.

Subsequently, 31 ERDEG indicators were selected using the random forest method, and LASSO regression analysis was performed to establish the LASSO risk model. By constructing the LASSO regression model diagram (**Figure 5D**) and LASSO variable trajectory diagram (**Figure 5E**), a graphical representation of the results of the LASSO regression analysis was realized. The study revealed that the LASSO risk assessment model comprised 10 ERDEGs elements: *TPP2*, *AKR1B1*, *CD59*, P*ARK7*, *PLXNB2*, *HLA-B*, *FAH*, *NDST1*, *SCARB1*, and *HNRNPA2B1*. These specific sequences were selected as the focus of the study in a follow-up inquiry, and a forest pattern map showing the transitions of these sequences was created (**Figure 5F**).

### 3.6 Key genes to construct the diagnostic logistic regression model

To obtain the diagnostic model for PPD, we used 10 key genes (*TPP2*, *AKR1B1*, *CD59*, P*ARK7*, *PLXNB2*, *HLA-B*, *FAH*, *NDST1*, *SCARB1*, and *HNRNPA2B1*) to calculate the relative contribution weights of these genes to the risk of diseases. Subsequently, using the RiskScore formula, the expression and coefficients of the 10 major genes in the GSE45603 dataset were included in the calculation to determine the RiskScore value for each sample. Then, according to the medium-risk level, the PPD group was subdivided into a lower-risk group and a higher-risk group. The risk assessment formula was as follows:


$$\begin{array}{l} \text{RiskScore}\;=\;-10.9487\;*\text{C}+4.5673\;*\text{D}+0.4049\;*\text{E}+1.8916\;*\text{F}\\ \;-2.1980\;*\text{G}-2.0310\;*\text{H}-0.4907\;*\text{I}-2.0357\;*\text{J}\\ \;-1.1504\;*\text{K}-0.9922\;*\text{L} \end{array}$$

**Figure 6:**
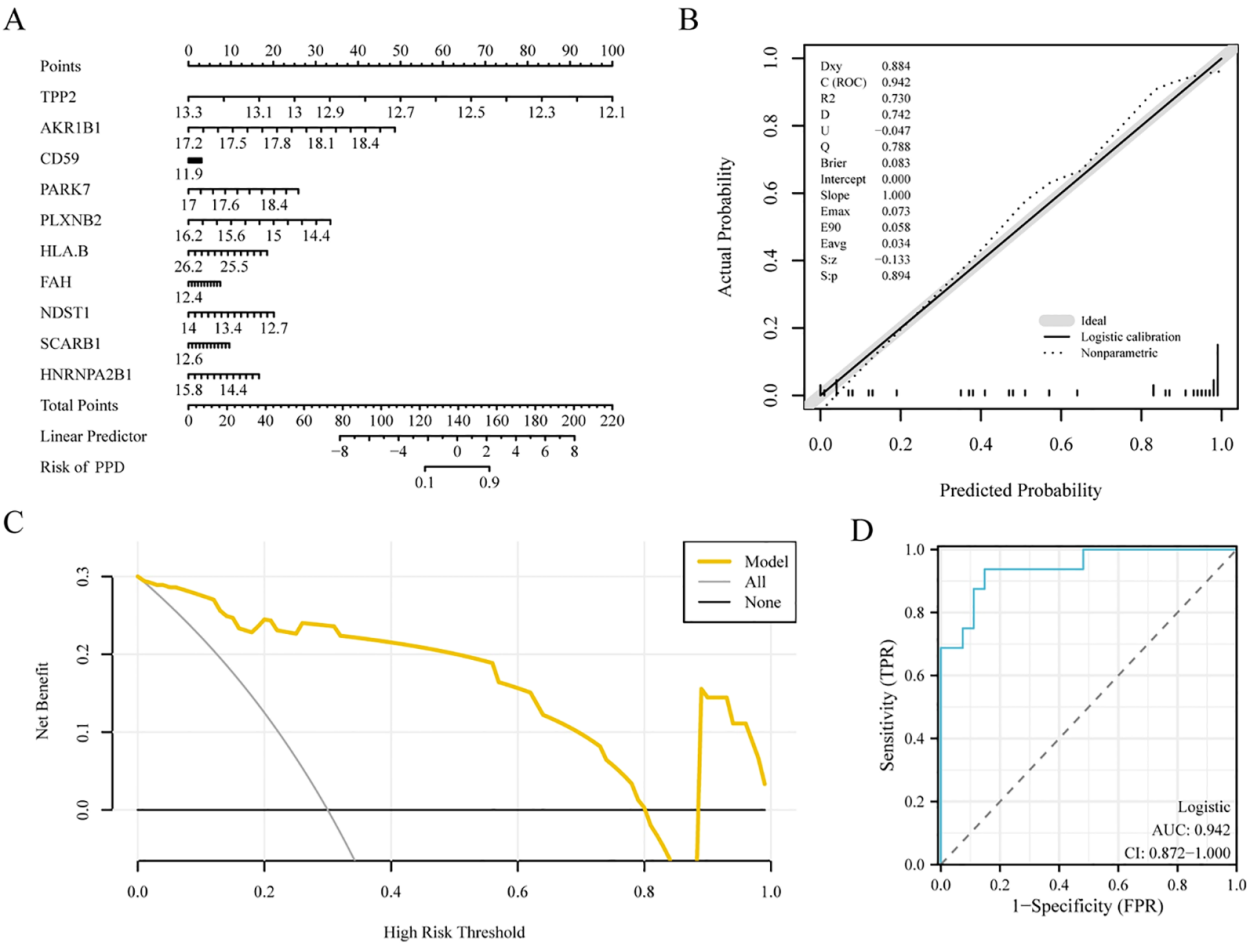
Key genes to construct diagnostic logistic regression model. **(A)** Nomogram of key genes in the diagnostic multivariate logistic model based on dataset GSE45603. **(B)** Calibration nomogram of key genes for the diagnostic multivariate logistic model based on the GSE45603 dataset. **(C)** Decision curve analysis (DCA) plot of the key genes of the diagnostic multivariate logistic model based on dataset GSE45603. **(D)** Diagnostic receiver operating characteristic curve (ROC) curve of the risk score of the diagnostic multivariate logistic model (Riskscore) in data set GSE45603. The multivariate Logistic model was constructed based on ten genes. The ordinate of the DCA plot is the net benefit, and the abscissa is the probability threshold or threshold probability. The closer the area under the curve (AUC) is to 1 on the ROC curve, the better the diagnostic performance. When the AUC was above 0.9, the accuracy was high. DCA, Decision curve analysis; ROC, Receiver operating characteristic curve; AUC, Area under the curve; PPD, Postpartum depression.

We then drew a nomogram (**Figure 6A**) to show the connections between the 10 key genes, and the expression of *TPP2* contributed the most in the multivariate logistic model.

Calibration analyses were performed and calibration charts were prepared to evaluate the precision and discriminative power of the multiple logistic regression model. The ability of the model to predict the actual outcome was analyzed by examining the alignment of the observed and estimated probabilities in the graph for different scenarios (**Figure 6B**). The calibration plot of the multiple logistic regression model revealed that the calibration curve indicated by the dotted line aligned with the main diagonal of the perfect model, indicating that the model was accurate and discriminative.

Furthermore, using dataset GSE45603, we implemented DCA to evaluate the performance of the multivariate logic diagnostic model for PPD (**Figure 6C**). The results showed that the linear stability of the model was superior to those of the overall and ineffective models within certain limits, and the net benefit was more significant, indicating the superiority of the model in diagnostic performance.

Finally, using the pROC package in R language software, we drew the ROC curve of the RiskScore index in dataset GSE45603 to evaluate the accuracy of the multiple logistic regression model in determining PPD. The multivariate Logistic model was constructed based on ten genes. The plot of the ROC curve indicated the high degree of accuracy of the multivariate logistic regression model for disease identification (**Figure 6D**, AUC = 0.942).

### 3.7 Validation of expression disparities and analysis of functional similarity among pivotal genes

**Figure 7:**
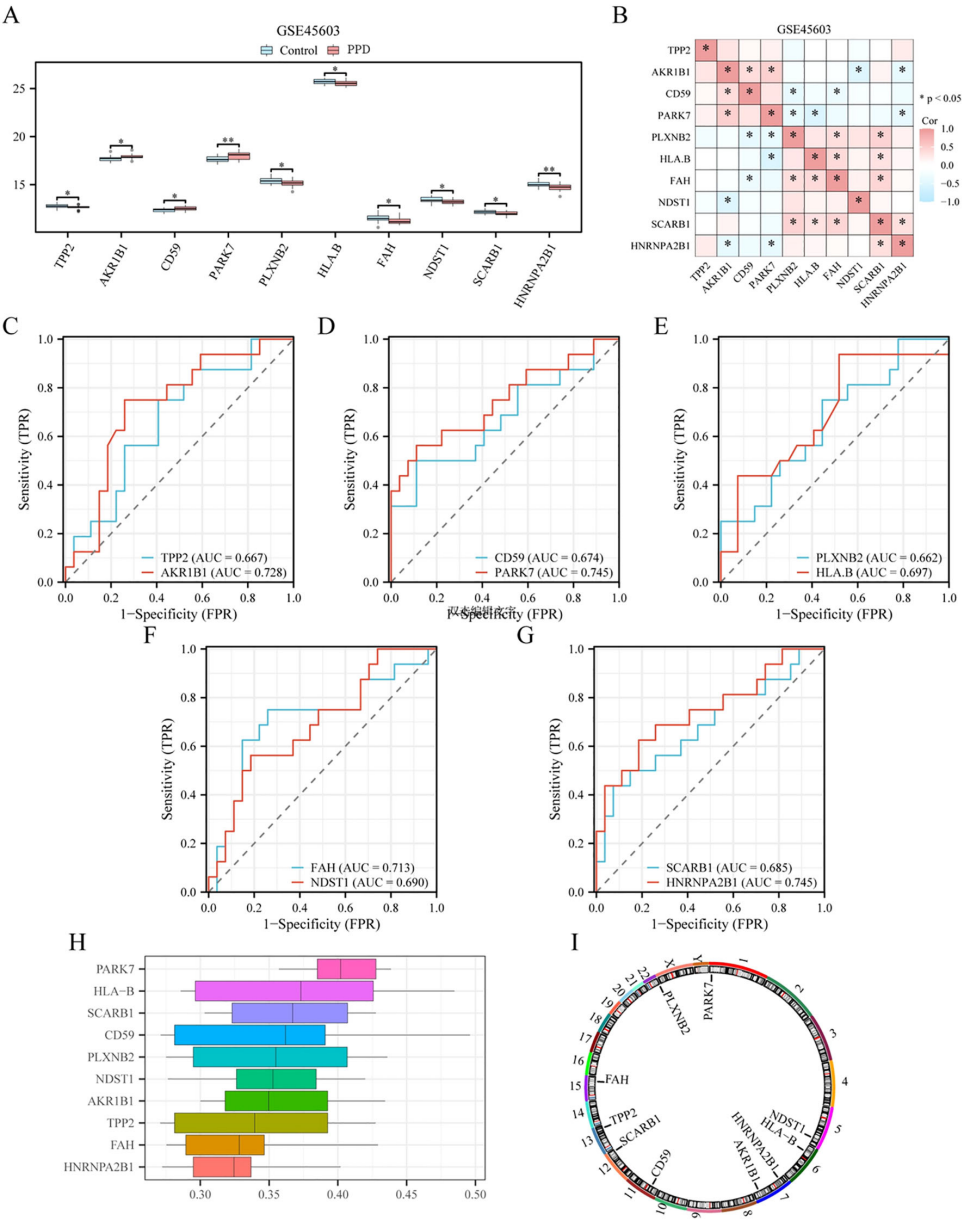
Expression difference verification and functional similarity analysis of key genes. **(A)** Group comparison diagram of key gene expression differences between different groups of postpartum depression (PPD)/Control) in dataset GSE45603. **(B)** Heat map of correlation between key genes in dataset GSE45603. **(C–G)**. Key genes: TPP2 **(C)**, AKR1B1 **(C)**, CD59 **(D)**, PARK7 **(D),** PLXNB2 **(E)**, HLA-B **(E)**, FAH **(F)**, NDST1 **(F)**, SCARB1 **(G),** ROC curve of HNRNPA2B1 **(G)** between different groups (PPD/Control) in dataset GSE45603. **(H)**. Plot of functional similarity of key genes. **(I)** Chromosomal mapping of key genes. * is equivalent to a p-value < 0.05, which is statistically significant; ** is equivalent to a p-value < 0.01 and is highly significant. The closer the area under the curve (AUC) in the receiver operating characteristic (ROC) curve is to 1, the better the diagnostic effect. When AUC was between 0.5 and 0.7, the accuracy was low. When AUC was between 0.7 and 0.9, it had a certain accuracy. Correlation intensity is 0.5 or lessr< 0.8: moderate; 0.3 or lessr< 0.5: low correlation. DCA, Decision curve analysis; ROC, Receiver operating characteristic curve; AUC, Area under the curve; PPD, Postpartum depression.

To confirm the disparities among the 10 pivotal genes (*TPP2*, *AKR1B1*, *CD59*, P*ARK7*, *PLXNB2*, *HLA-B*, *FAH*, *NDST1*, *SCARB1*, and *HNRNPA2B1*) in different groups (PPD/Control) of dataset GSE45603. Using the transcriptional profiles of these 10 crucial genes across various cohorts (PPD/control) of dataset GSE45603, the Wilcoxon rank-sum test was used to analyze the expression differences of the 10 key genes in different groups (PPD/Control) of dataset GSE45603, and the expression difference analysis results were displayed by grouping violin plots (**Figure 7A**). According to the grouped violin plot, two pivotal genes (*PARK7* and *HNRNPA2B1*) exhibited strong statistical significance (p < 0.01).

Subsequently, we conducted a correlation analysis and generated a correlation heatmap for the expression levels of 10 crucial genes within dataset GSE45603 (**Figure 7B**). This analysis revealed that *AKR1B1* and *PARK7* exhibited the highest positive correlation (r = 0.47, p < 0.05), whereas *HLA-B* and *PARK7* showed the most significant negative correlation (r = -0.56, p < 0.05).

To determine the diagnostic performance of 10 key genes (*TPP2*, *AKR1B1*, *CD59*, *PARK7*, *PLXNB2*, *HLA-B*, *FAH*, *NDST1*, *SCARB1*, and *HNRNPA2B1*) for PPD in dataset GSE45603, we drew the ROC curves of 10 key genes in the GSE45603 disease control group (PPD/Control); the results are presented in **Figures 7C–G**). According to the ROC curve, in dataset GSE45603, *TPP2* (AUC = 0.667, **Figure 7C**), *CD59* (AUC = 0.674, **Figure 7D**), *PLXNB 2* (AUC = 0.662, **Figure 7E**), *HLA-B* (AUC = 0.697, **Figure 7E**), *NDST1* (AUC = 0.690, **Figure 7F**), *SCARB1* (AUC = 0.685, **Figure 7G**) had low accuracy in PPD diagnosis. *AKR1B1* (AUC = 0.728, **Figure 7C**), *PARK7* (AUC = 0.745, **Figure 7D**), *FAH* (AUC = 0.713, **Figure 7F**), and *HNRNPA2B1* (AUC = 0.745, **Figure 7G**) accurately diagnosed PPD.

To explore the functional relevance of the 10 core genes, we used the GOSemSim toolkit to estimate the GO semantic similarity of these genes in terms of BP, CC, and MF and then calculated their average geometric values at these three levels to obtain a comprehensive score. The similarity scores between each core gene and other core genes were averaged and ranked in descending order. The data from the functional similarity analysis are presented as box plots using the ggplot toolkit (**Figure 7H**). The graph reveals a high degree of functional similarity between *PARK7* and several core genes. Finally, we constructed a chromosome mapping chart (**Figure 7I**), showing the distribution of 10 major loci at the chromosomal level.

### 3.8 Analysis of logistic risk score for the high and low group GSEA enrichment

**Figure 8:**
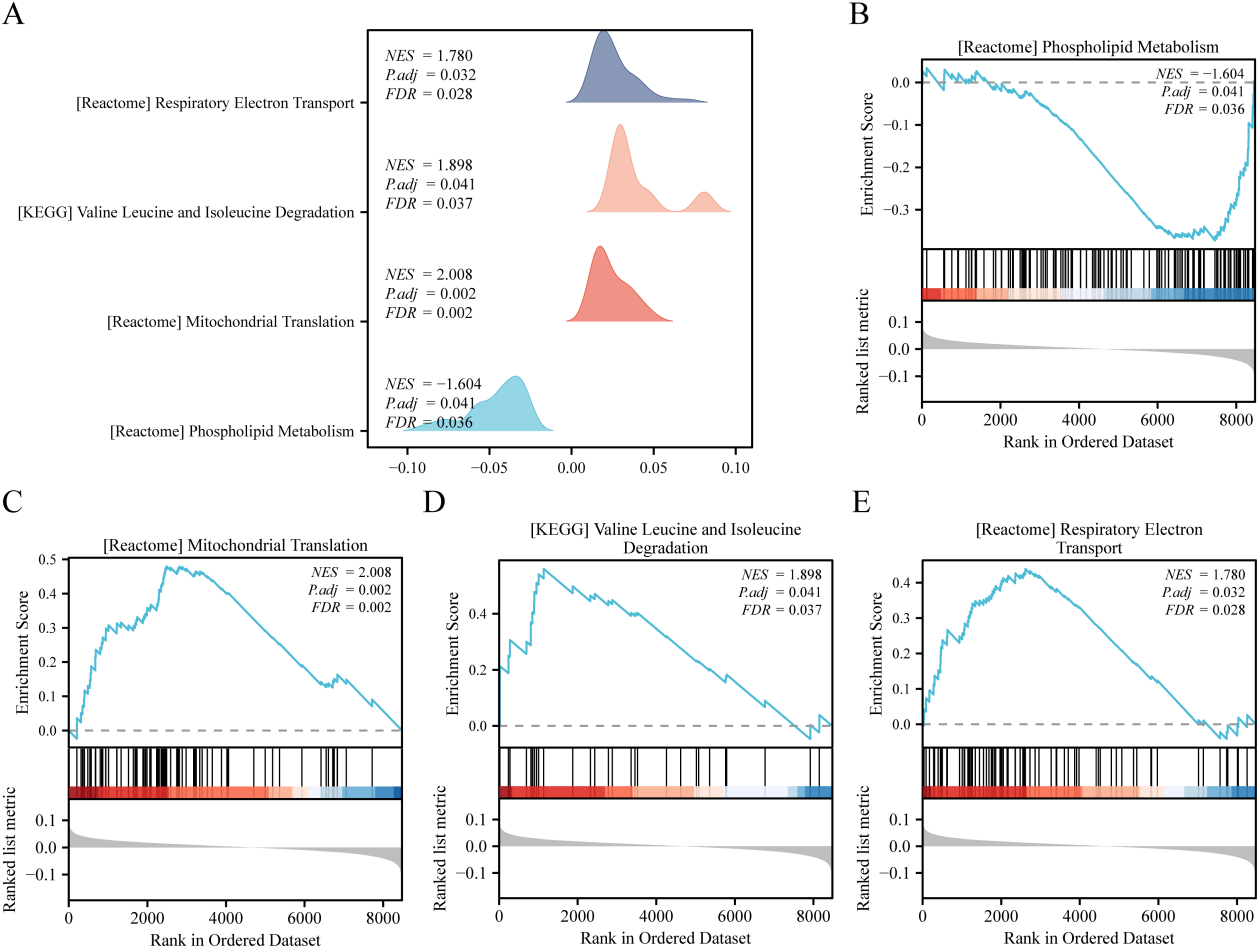
GSEA based on high and low logistic risk score groups. **(A)**, Gene set enrichment analysis (GSEA) of genes between different groups (Low/High) in dataset GSE45603 showed the main four biological characteristics of mountain plots. B-e. The genes in dataset GSE45603 were significantly enriched in the REACTOME_PHOSPHOLIPID_METABOLISM **(B)**, REACTOME_MITOCHONDRIAL_TRANSLATION **(C)**, KEGG_VALINE_LEUCINE_AND_ISOLEUCINE_DEGRADATION **(D)**, and REACTOME_RESPIRATORY_ELECTRON_TRANSPORT **(E)** pathways. The screening criteria of GSEA were p-value adj < 0.05 and false-discovery rate value (q value) < 0.25, and the Benjamini-Hochberg method was used for p-value correction. GSEA, Gene set enrichment analysis; PPD, Postpartum depression; BH, Benjamini-Hochberg.

To investigate the correlation between the expression levels of genes in GSE45603 and the high or low-risk classifications of the group PPD, we used the GSEA method to analyze the expression of 11,819 genes in different high- and low-risk ratings (low or high) of the PPD group and the biological processes involved. The criteria for significant enrichment were set at a p-value adj < 0.05 and a false discovery rate value (q value) < 0.25. Significantly enriched pathways screened using GSE45603 are depicted using a mountain plot (**Figure 8A**). Finally, the relevant genes in various groups with low or high GSE45603 values showed significant clustering in the phospholipid biosynthesis pathway (**Figure 8B**), mitochondrial translation (**Figure 8C**), valine, leucine, and isoleucine degradation (**Figure 8D**), respiratory electron transport (**Figure 8E**), and other pathways (**Figures 8B–E**, **Supplementary Table S3**).

### 3.9 GSVA based on logistic risk-score grouping

This study aimed to investigate the difference between H.all.v7.4. symbols. For the gmt gene set in dataset GSE45603 between the low- and high-risk-score groups of patients with PPD, we performed GSVA on all genes in dataset GSE45603.

**Figure 9:**
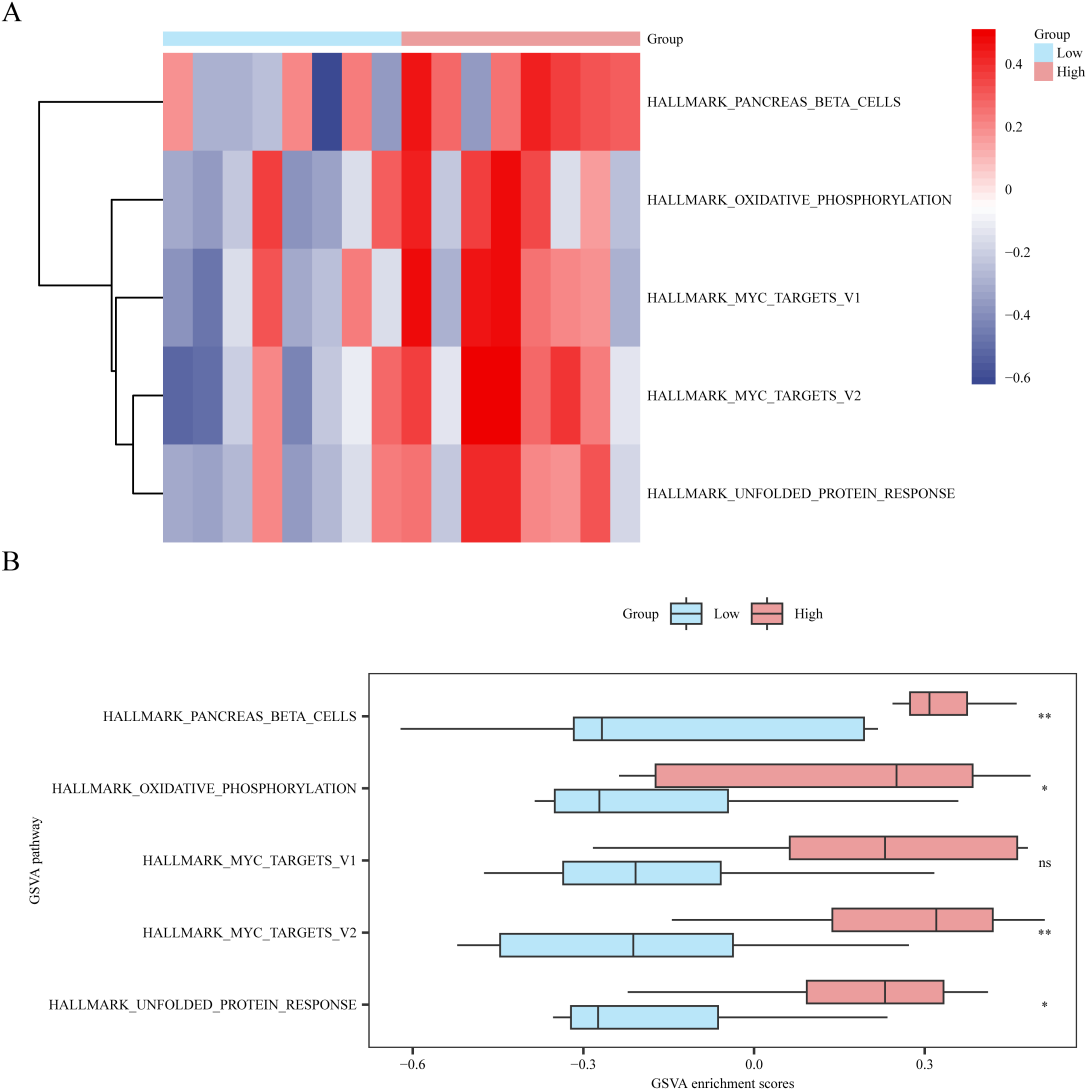
Gene set variation analysis based on high and low logistic risk score groups. **(A, B)** Complex numerical heatmap **(A)** and group comparison boxplot **(B)** of gene set variation analysis results in the dataset GSE45603 risk score high and low groups (Low/High). ns is equivalent to a p-value ≥ 0.05, which is not significant; * is equivalent to a p-value < 0.05 and is significant. Light blue represents the low-risk group (Low), and light red represents the high-risk group (High). The screening criteria for gene set variation analysis (GSVA) was p value < 0.05, the p-value correction method was p value < 0.05, and the p-value correction method was Benjamini-Hochberg (BH) method. GSVA, Gene set variation analysis. BH, Benjamini-Hochberg (BH). GSVA, Gene set variation analysis. BH, Benjamini-Hochberg.

According to the GSVA results, the differential expression of five pathways with significant enrichment (p < 0.05) (**Supplementary Table S4**) between the high- and low-risk score groups (low or high) was analyzed and visualized using a heat map (**Figure 9A**) and a group comparison map (**Figure 9B**). Four pathways showed statistical significance in distinguishing between the high- and low-risk-score groups (p-value < 0.05) after analysis. The pathways included MYC target V2, pancreatic beta cells, unfolded protein response, and oxidative phosphorylation.

### 3.10 ssGSEA immune infiltration analysis based on logistic risk score grouping

**Figure 10:**
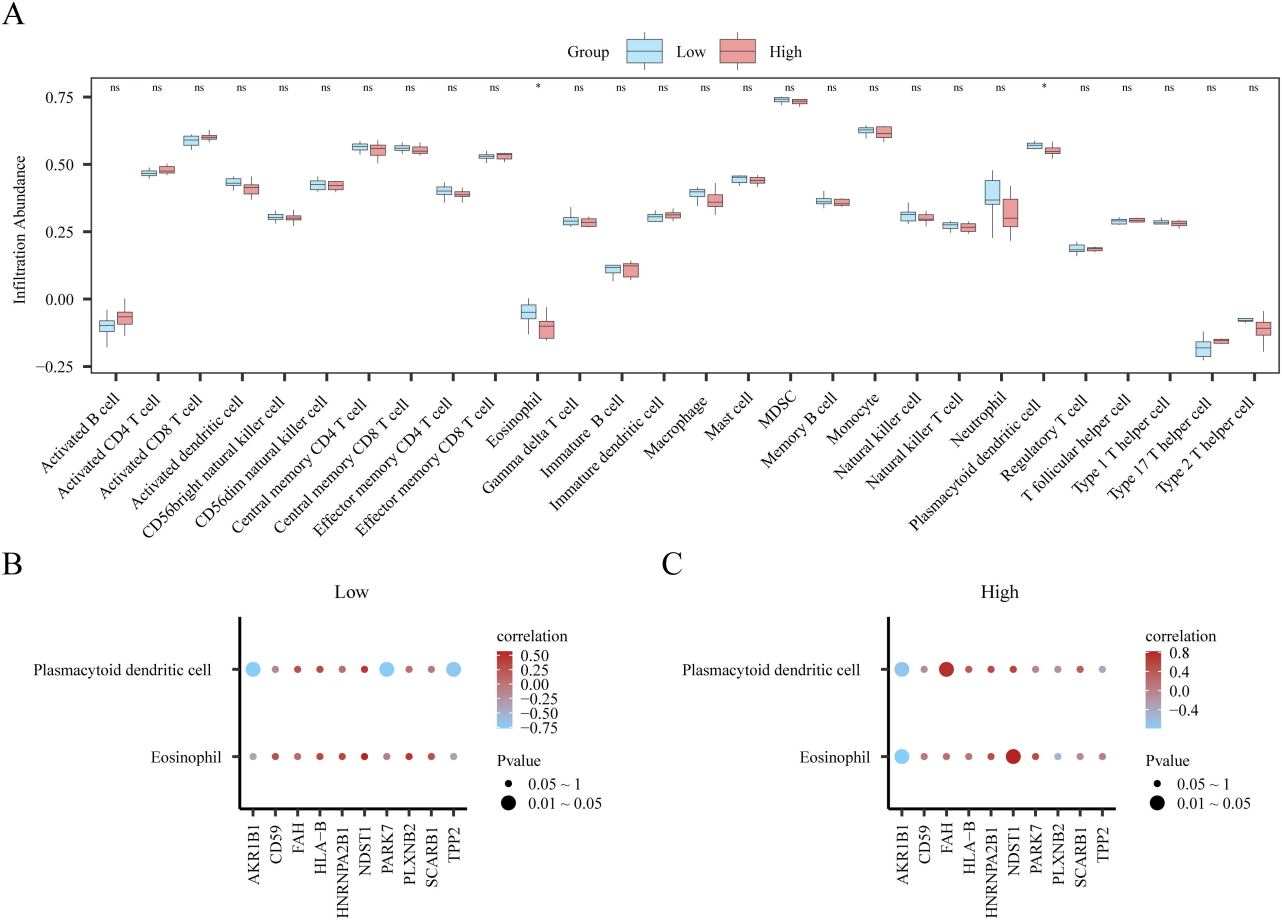
Single-sample gene set enrichment analysis (ssGSEA) immune infiltration analysis based on high and low Logistic risk score groups. **(A)** Group comparison of ssGSEA immune infiltration analysis results between low/high-risk groups of patients with PPD in dataset GSE45603. **(B)** Heat map of correlation between immune cells with significant differences and key genes in the group comparison plot **(A)** of PPD low-risk group (Low) samples in the GSE45603 dataset. **(C)** Heat map of correlation between immune cells with significant differences and key genes in group comparison map **(A)** in PPD high-risk group (High) samples of GSE45603 data set. ns is equivalent to p value ≥ 0.05, which is not significant. * is equivalent to a p-value < 0.05, which is significant. Light blue represents the low-risk group (Low), and light red represents the high-risk group (High). Red is a positive correlation, blue is a negative correlation, and the depth of the color represents the strength of the correlation. PPD, Postpartum depression. ssGSEA, Single-sample gene-set enrichment analysis.

We used ssGSEA to quantify the infiltration levels of 28 immune cell types in low- and high-risk patient subsets from dataset GSE45603. The Mann–Whitney U test was conducted to evaluate the disparities in the accumulation of the 28 immune cell types between the two risk stratifications (high and low). The results are presented as a group comparison plot (**Figure 10A**). The survey discovered that for dataset GSE45603, immune cells, immature B cells, and Tregs, the infiltrating abundance of T follicular helper (Tfh) cells showed significant discrimination among different risk groups (*p* < 0.05).

Next, we used the “Pearson” method to calculate the eosinophil, plasmacytoid dendritic cell, and immune cell counts in low- or high-risk PPD samples in the GSE45603 dataset. The degree of enrichment of eosinophils and plasmacytoid dendritic cells was significantly correlated with the expression levels of 10 key genes (*TPP2*, *AKR1B1*, *CD59*, *PARK7*, *PLXNB2*, *HLA-B*, *FAH*, *NDST1*, *SCARB1* and *HNRNPA2B1*), and this finding was visualized by a correlation heatmap (**Figures 10B, C**). Samples from the low-risk group (low) in the GSE45603 dataset showed a significant positive correlation between eosinophils and *NDST1* expression in immune cells (r = 0.568, *p* < 0.05). In an immune cell study of high-risk eosinophil samples, a significant positive correlation with the index *NDST1* was observed (r = 0.822, p < 0.05).

### 3.11 Regulatory network analysis of key genes

**Figure 11:**
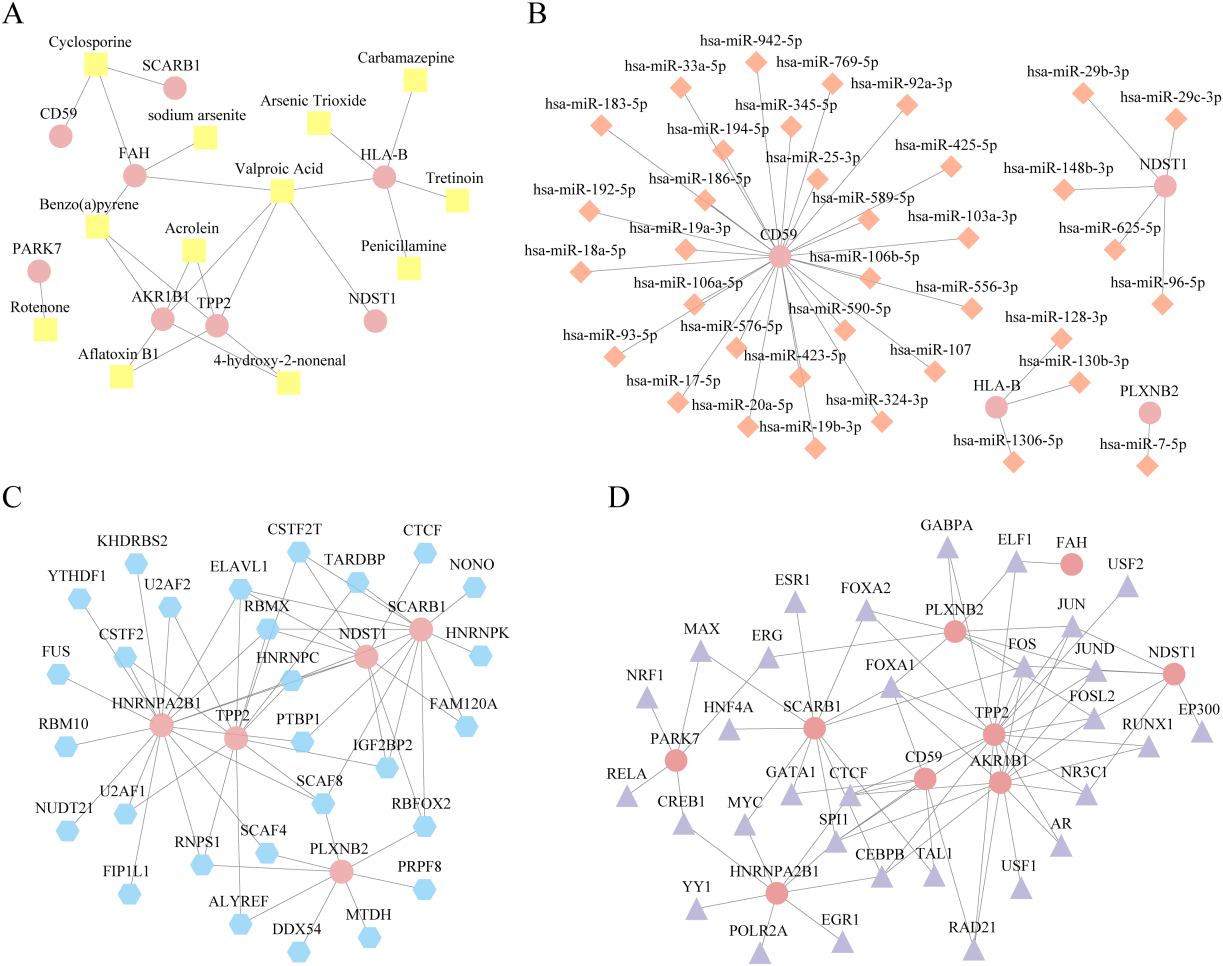
Regulatory network analysis of key genes. **(A)** Diagram of microRNA (mRNA)-drugs interaction network of key genes, pink oval blocks are mrnas and yellow square blocks are drugs. **(B)** mRNA-mirna interaction network diagram of key genes, pink oval block is mRNA, orange diamond block is miRNA. **(C)** mRNA- RNA binding protein (RBP) interaction network diagram of key genes. The pink oval block represents mRNA, and the blue polygon block represents RBP. **(D)** mRNA-RBP interaction network diagram of key genes. The pink oval blocks are mrnas, and purple diamonds are transcription factors (TFs). TF, Transcription factors; RBP, RNA binding protein; TFS, transcription factors.

We used the CTD database to predict the regulatory network of 10 key genes in order to conduct relevant targeted regulation in the future (**Figure 11**).

## 4 Discussion

Symptoms such as emotional depression, lack of interest or satisfaction, and reduced vitality form a core symptom group that co-occur with other psychological manifestations and is classified as a syndrome of mental illness and somatic symptoms. This condition usually manifests within 4–6 weeks (34). PPD following childbirth adversely affects mothers, increases suicide risk, and profoundly influences the health and growth of babies and children. This includes a heightened risk of infant mortality, stunted growth and development in children, and possible autism spectrum disorder escalation (35). Current diagnostic methods for PPD are limited and often subjective, relying heavily on standardized questionnaires, including scales and clinical evaluations (36). These methods are time-consuming and prone to bias, leading to potential misdiagnosis and delayed treatment. Moreover, treatment options for PPD, including pharmacotherapy and psychotherapy, are not always effective and can have side effects, further emphasizing the need for more precise diagnostic tools and targeted therapies (37). Biomarkers can objectively measure and evaluate biological processes, drug reaction indices, pathological processes, disease diagnosis, treatment monitoring, and prognostic assessments, which play an important role (38). Identifying and measuring biomarkers associated with PPD can improve the accuracy and timeliness of diagnosis for effective management and treatment of the disease (39). Biomarkers in the diagnosis and treatment of PPD have important application value in monitoring; however, the underlying molecular mechanism remains unclear. Further studies are needed to determine the biomarkers and develop effective diagnosis and treatment strategies and precision medical support for PPD.

Exosomes are a subtype of extracellular vesicles that possess a dual lipid membrane architecture, function as reliable data transporters, and facilitate unrestricted movement between the peripheral blood flow and the central nervous system. According to the definition of MISEV, “exosome” refers to EVs released from the interior of the cell through multivesicular bodies (MVBS). Its significance extends beyond bodily functions and encompasses a multitude of diseases (40). Furthermore, the body emits bioactive elements such as nucleic acids and proteins in response to alterations in the central nervous system operations, leading to a corresponding dynamic adaptation (41). Several studies have established the crucial functions of exosomes in the development and progression of depression. For instance, external secretion of the body by depression can result in depressive behaviors in mice, a process that encompasses the miR-139-5p regulation of neural functions (42). However, research on whether exosomes can be used as specific diagnostic biomarkers for PPD is lacking. This will help to explore further relevant therapeutic targets for postpartum depressive disorders, particularly PPD. This highlights the urgent need for extensive validation to enhance our understanding of the role of exosomes in PPD.

Analysis of functional annotation enrichment encompassing the GO and KEGG pathways provided a comprehensive overview of the BPs, CCs, and MFs associated with the identified ERDEGs. Notably, the enriched GO terms included those related to synaptic signaling, hormonal changes, and inflammatory responses, which aligns with previous findings suggesting that neurotransmitter imbalances, hormonal fluctuations post-delivery, and immune system dysregulation could contribute to PPD pathophysiology (43). Furthermore, KEGG pathway analysis emphasized the significance of pathways involving neuroactive ligand-receptor and cytokine-cytokine receptor interactions, supporting the hypothesis that altered communication within neural circuits and between immune signaling molecules may be crucial in PPD development (44).

The identification of key genes through logistic regression, random forest, and LASSO regression analyses underscores the robustness of our approach in identifying critical molecular players in PPD. The selection of 10 key genes (*TPP2, AKR1B1, CD59, PARK7, PLXNB2, HLA-B, FAH, NDST1, SCARB1, and HNRNPA2B1*) from the initial 44 ERDEGs highlighted their potential as biomarkers for PPD. Because both PARK7 and HNRNPA2B1 showed better diagnostic performance than the other genes in the area under the ROC curve (AUC) assessment (AUC = 0.745) and also had a strong influence in terms of statistical significance (P value), In addition, there is sufficient biological evidence in the postpartum depression (PPD) literature to support its importance in the disease mechanism, especially its key role in neuroprotection and cell signaling. So PARK7 and HNRNPA2B1 were selected as the most important genes. *PARK7* (Parkinsonism-associated deglycase), also known as DJ-1, is a versatile protein that participates in response to oxidative stress, regulation of mitochondrial activities, and safeguarding of neural cells. The altered regulation of this process has been associated with a range of neurodegenerative conditions, such as Parkinson’s disease and depression (45). Notably, PARK7, previously linked to oxidative stress in Parkinson’s disease, here correlated strongly with PPD risk, suggesting its role may extend to perinatal mood disorders via exosome-mediated pathways. *HNRNPA2B1* is involved in RNA processing, transport, and stability. The dysregulation of this gene has been associated with various neurodegenerative and neuropsychiatric disorders (46). Subsequent validation of these genes through differential expression and ROC curve analyses confirmed their diagnostic utility. Notably, *PARK7* and *HNRNPA2B1* showed the highest statistical significance, suggesting their prominent roles in PPD. *AKR1B1* is involved in the polyol pathway that converts glucose to sorbitol and has been implicated in oxidative stress and inflammatory responses, both of which are relevant to the pathophysiology of PPD (47). Elevated *AKR1B1* expression may exacerbate oxidative stress and contribute to neuronal damage and mood disorders. Our findings indicated that *AKR1B1* was significantly upregulated in patients with PPD, consistent with its proposed role in neuroinflammation and oxidative stress. Correlation analysis further revealed the intricate relationships among these key genes, with *AKR1B1* and *PARK7* showing the strongest positive correlations. These findings validate the diagnostic capability of these critical genes and provide a foundation for future functional studies to elucidate their roles in PPD.

The diagnostic logistic regression model using the 10 pivotal genes exhibited robust diagnostic efficacy for PPD, as evidenced by an AUC of 0.942. Calibration curve analysis confirmed the reliability of the model, with the predicted probabilities closely aligned with the actual outcomes. DCA confirmed the practical clinical value of the model, showing a high net benefit across a range of threshold probabilities. The nomogram provides a user-friendly tool for clinicians to perform risk assessments derived from the transcriptional activity of pivotal genes. These findings highlight the potential of the logistic regression model as a dependable diagnostic method for PPD, contributing to the timely identification and management of the condition. This multi-analyte approach significantly outperforms single biomarkers, aligning with Rathi who advocated combinatorial signatures for complex disorders like PPD (39).

GSEA indicated that genes within the high- and low-risk categories were prominently involved in pathways, such as phospholipid metabolism and mitochondrial translation. Phospholipid metabolism is essential for maintaining the integrity of cellular membranes and signaling, which is fundamental for the proper functioning of neurons and synaptic plasticity. Dysregulation of this pathway may contribute to the development of PPD by influencing neuronal communication and brain function. Mitochondrial translation is critical for the production of mitochondrial proteins that are crucial for energy production and metabolism. Mitochondrial dysfunction is associated with various neuropsychiatric conditions, including depression, indicating that mitochondrial malfunction may be involved in PPD pathogenesis (48). GSVA revealed several notable pathways, including those related to MYC targets, pancreatic beta cells, the unfolded protein response, and oxidative phosphorylation. The MYC pathway is involved in controlling cell growth, division, and programmed cell death. Its dysregulation is associated with various mental health conditions (49). The role of pancreatic beta cells in PPD is intriguing, hinting at a possible connection between metabolic activity and mood modulation, which may be mediated through insulin signaling pathways (50). The unfolded protein response is a biological response activated by the endoplasmic reticulum under stress and is linked to neuroinflammation and neurodegeneration. These processes are considered relevant to the onset of depressive symptoms (51). Oxidative phosphorylation is the main process through which the mitochondria is involved in the synthesis of adenosine triphosphate), the cell’s main energy provider. Any disruption in this process can result in diminished energy levels within neurons, which has been implicated in the manifestation of depressive symptoms (52). The discovery of these molecular pathways offers more profound insights into the underlying mechanisms of PPD and pinpoints areas for potential therapeutic intervention. The substantial enrichment of these pathways in both high- and low-risk groups suggests that they could function as indicators for diagnosing and predicting the outcome of PPD, contributing to the advancement of targeted and effective treatment approaches.

Evaluating immune cell penetration using ssGSEA revealed notable variations in the levels of immature B cells, Tregs, and Tfh cells between individuals classified as high and low risk for PPD. Immature B cells are pivotal in the adaptive immune response and serve as precursors of mature B cells that produce antibodies. Abnormalities in B-cell development and function have been associated with various mental health conditions, indicating a possible connection to PPD (53). Tregs play pivotal roles in maintaining immune homeostasis and preventing autoimmune responses. The altered abundance of Tregs may reflect a compromised ability to regulate immune responses, potentially leading to increased neuroinflammation and mood disturbances (54). Tfh cells are crucial for forming germinal centers and producing highly affine antibodies. These cells also modulate immune responses and have been linked to the development of autoimmune disorders and persistent inflammation (55). The increased levels of immature B cells and Tfh cells observed in high-risk populations suggest persistent immune activation or compromised immune responses, which may exacerbate PPD symptoms, highlighting the role of the immune system in PPD and identifying potential therapeutic targets. Our observation of altered immune infiltration extends findings by Konstantinou et al, who proposed immune-inflammatory pathways in PPD. Here, ERGs may modulate this response via exosomes, offering a mechanistic link not previously explored. More targeted and effective treatments can be developed by comprehending the precise functions and mechanisms of these immune cells in relation to PPD. Additionally, incorporating immune cell profiling into diagnostic models could improve the accuracy of PPD diagnosis and allow personalized treatment plans. Future research should delve into the specific mechanisms by which these immune cells impact PPD and investigate their potential as biomarkers for early detection and intervention.

Although our study demonstrated good predictive performance and high reliability and stability of the results, the study had some limitations. Our findings are mainly based on bioinformatics analysis of publicly available transcriptome databases and have not yet included experimental validation such as *in vitro* or *in vivo* functional studies. We have fully recognized this limitation, and we plan to conduct further relevant experiments in the future to verify the biological role of the screened key genes in postpartum depression, so as to improve the translational value and reliability of the research conclusions. In this study, we screened and verified exosome-related gene features for PPD diagnosis by bioinformatics methods, and the diagnostic model showed high accuracy on the basis of existing samples. It should be pointed out that research is currently based on a single dataset (GSE45603), the sample size of this study is limited (43 cases in total), which is mainly limited by the accessibility of clinical data in public databases and ethical constraints, validation of independent external cohorts or clinical samples was not performed. Multiple algorithms and cross-validation have been used to improve the robustness of the results, but the limited sample size may still affect the extrapolation and power of the conclusions. Although we tried our best to correct for batch effects during the analysis, potential batch bias cannot be completely ruled out. In the future, the sample size and multi-center clinical validation will be further expanded to enhance the reliability and clinical application value of the research conclusions.

In summary, this study, through in-depth bioinformatics exploration, reveals the possible functions of key cellular receptor agonists (ERGs) in PPD. The constructed diagnostic model demonstrated high accuracy, as evidenced by ROC curve analysis. By evaluating functional enrichment and immune penetration, we gained valuable insights into biological processes and the immune system. Despite the limitations, the study’s findings lay a promising foundation for future academic exploration and potential applications in medical practice, including the development of new diagnostic methods and therapies.

## Data Availability

The original contributions presented in the study are included in the article/**Supplementary Material**. Further inquiries can be directed to the corresponding author.
