# Supplementary material for: Gene signatures associated with exosomes as diagnostic markers of postpartum depression and their role in immune infiltration
Source: Front Endocrinol (Lausanne). 2025 Jul 17;16:1542327. doi: 10.3389/fendo.2025.1542327 (PMC12310459; doi:10.3389/fendo.2025.1542327)
Supplement: Supplementary file 1 [file Table1.docx]

### Table 1. Postpartum depression data set information list.

|  | **GSE45603** |
| --- | --- |
| Platform | GPL10558 |
| Species | Homo sapiens |
| Tissue | Peripheral blood |
| Samples in PD group | 16 |
| Samples in Control group | 27 |
| Reference | - |

PD，Postpartum depression；GEO，Gene expression omnibus。
