# Supplementary material for: Gene signatures associated with exosomes as diagnostic markers of postpartum depression and their role in immune infiltration
Source: Front Endocrinol (Lausanne). 2025 Jul 17;16:1542327. doi: 10.3389/fendo.2025.1542327 (PMC12310459; doi:10.3389/fendo.2025.1542327)
Supplement: Supplementary file 2 [file Table2.docx]

### Table 2. GO enrichment Analysis results of ERDEGs.

| ONTOLOGY | ID | Description | GeneRatio | BgRatio | pvalue | p.adjust | qvalue |
| --- | --- | --- | --- | --- | --- | --- | --- |
| BP | GO:0006417 | regulation of translation | 9/42 | 463/18800 | 6.6798E-07 | 0.00058689 | 0.00043637 |
| BP | GO:0034470 | ncRNA processing | 7/42 | 424/18800 | 3.8525E-05 | 0.00555446 | 0.00412991 |
| BP | GO:0022613 | ribonucleoprotein complex biogenesis | 7/42 | 448/18800 | 5.4595E-05 | 0.00561994 | 0.00417859 |
| BP | GO:0010498 | proteasomal protein catabolic process | 7/42 | 496/18800 | 0.00010334 | 0.00902679 | 0.00671169 |
| BP | GO:0045727 | positive regulation of translation | 6/42 | 141/18800 | 6.7188E-07 | 0.00058689 | 0.00043637 |
| CC | GO:0005925 | focal adhesion | 8/44 | 419/19594 | 3.6928E-06 | 0.00020082 | 0.0001307 |
| CC | GO:0030055 | cell-substrate junction | 8/44 | 428/19594 | 4.3187E-06 | 0.00020082 | 0.0001307 |
| CC | GO:0005840 | ribosome | 6/44 | 229/19594 | 1.1624E-05 | 0.00042004 | 0.00027337 |
| CC | GO:0030139 | endocytic vesicle | 6/44 | 342/19594 | 0.00010919 | 0.0022565 | 0.00146858 |
| CC | GO:0022626 | cytosolic ribosome | 5/44 | 102/19594 | 3.201E-06 | 0.00020082 | 0.0001307 |
| MF | GO:0003735 | structural constituent of ribosome | 5/44 | 181/18410 | 6.9178E-05 | 0.01452748 | 0.01114138 |
| MF | GO:0003924 | GTPase activity | 5/44 | 336/18410 | 0.00119053 | 0.04166857 | 0.03195634 |
| MF | GO:0019843 | rRNA binding | 3/44 | 66/18410 | 0.00052471 | 0.03672964 | 0.0281686 |
| MF | GO:0019003 | GDP binding | 3/44 | 73/18410 | 0.00070491 | 0.03700796 | 0.02838204 |
| MF | GO:0004298 | threonine-type endopeptidase activity | 2/44 | 14/18410 | 0.00049883 | 0.03672964 | 0.0281686 |
| KEGG | hsa05171 | Coronavirus disease - COVID-19 | 6/36 | 232/8164 | 0.00047032 | 0.04081828 | 0.03767362 |
| KEGG | hsa03010 | Ribosome | 5/36 | 158/8164 | 0.00059157 | 0.04081828 | 0.03767362 |

GO：Gene ontology；BP：Biological process；CC：Cellular component；MF：Molecular function。ERDEGs，Exosome related differentially expressed genes。
