# Supplementary material for: Gene signatures associated with exosomes as diagnostic markers of postpartum depression and their role in immune infiltration
Source: Front Endocrinol (Lausanne). 2025 Jul 17;16:1542327. doi: 10.3389/fendo.2025.1542327 (PMC12310459; doi:10.3389/fendo.2025.1542327)
Supplement: Supplementary file 3 [file Table3.docx]

### Table 3. GSEA enrichment analysis results of PD dataset GSE45603.

| ID | setSize | enrichmentScore | NES | pvalue | p.adjust | qvalue |
| --- | --- | --- | --- | --- | --- | --- |
| REACTOME_PHOSPHOLIPID_METABOLISM | 132 | -0.37257989 | -1.6036111 | 0.00076595 | 0.04054291 | 0.03606054 |
| REACTOME_MITOCHONDRIAL_TRANSLATION | 86 | 0.47980868 | 2.00794495 | 6.1712E-06 | 0.00196824 | 0.00175063 |
| KEGG_VALINE_LEUCINE_AND_ISOLEUCINE_DEGRADATION | 32 | 0.56024267 | 1.89816971 | 0.0008519 | 0.04125058 | 0.03668997 |
| REACTOME_RESPIRATORY_ELECTRON_TRANSPORT | 74 | 0.43918244 | 1.77972801 | 0.00046981 | 0.03155811 | 0.02806908 |

GSEA，Gene set enrichment analysis；PD，Postpartum depression。
