# Supplementary material for: Gene signatures associated with exosomes as diagnostic markers of postpartum depression and their role in immune infiltration
Source: Front Endocrinol (Lausanne). 2025 Jul 17;16:1542327. doi: 10.3389/fendo.2025.1542327 (PMC12310459; doi:10.3389/fendo.2025.1542327)
Supplement: Supplementary file 4 [file Table4.docx]

### Table 4. GSVA enrichment analysis results of PD dataset GSE45603.

| id | logFC | AveExpr | t | P.Value | adj.P.Val | B |
| --- | --- | --- | --- | --- | --- | --- |
| HALLMARK_MYC_TARGETS_V2 | -0.43686 | 0.030584 | -3.22137 | 0.001335 | 0.046629 | -1.12806 |
| HALLMARK_PANCREAS_BETA_CELLS | -0.40461 | 0.051803 | -3.12281 | 0.001865 | 0.046629 | -1.42231 |
| HALLMARK_UNFOLDED_PROTEIN_RESPONSE | -0.32993 | 0.005721 | -2.48037 | 0.013359 | 0.17544 | -3.11614 |
| HALLMARK_MYC_TARGETS_V1 | -0.33473 | 0.017825 | -2.46255 | 0.014035 | 0.17544 | -3.1576 |
| HALLMARK_OXIDATIVE_PHOSPHORYLATION | -0.29103 | 0.001408 | -2.2096 | 0.027456 | 0.274559 | -3.7137 |

GSVA，Gene set variation analysis；PD，Postpartum depression。
