# Supplementary material for: Gene signatures associated with exosomes as diagnostic markers of postpartum depression and their role in immune infiltration
Source: Front Endocrinol (Lausanne). 2025 Jul 17;16:1542327. doi: 10.3389/fendo.2025.1542327 (PMC12310459; doi:10.3389/fendo.2025.1542327)
Supplement: Supplementary file 5 [file Table5.docx]

### Table 5. mRNA-drugs interaction network nodes.

| mRNA | Drug |
| --- | --- |
| TPP2 | 4-hydroxy-2-nonenal |
| TPP2 | Acrolein |
| TPP2 | Aflatoxin B1 |
| TPP2 | Benzo(a)pyrene |
| TPP2 | Valproic Acid |
| AKR1B1 | 4-hydroxy-2-nonenal |
| AKR1B1 | Acrolein |
| AKR1B1 | Aflatoxin B1 |
| AKR1B1 | Benzo(a)pyrene |
| AKR1B1 | Valproic Acid |
| CD59 | Cyclosporine |
| FAH | Benzo(a)pyrene |
| FAH | Cyclosporine |
| FAH | sodium arsenite |
| FAH | Valproic Acid |
| HLA-B | Arsenic Trioxide |
| HLA-B | Carbamazepine |
| HLA-B | Penicillamine |
| HLA-B | Tretinoin |
| HLA-B | Valproic Acid |
| NDST1 | Valproic Acid |
| PARK7 | Rotenone |
| SCARB1 | Cyclosporine |
