# Supplementary material for: Gene signatures associated with exosomes as diagnostic markers of postpartum depression and their role in immune infiltration
Source: Front Endocrinol (Lausanne). 2025 Jul 17;16:1542327. doi: 10.3389/fendo.2025.1542327 (PMC12310459; doi:10.3389/fendo.2025.1542327)
Supplement: Supplementary file 6 [file Table6.docx]

### Table 6. mRNA-miRNA interaction network nodes.

| mRNA | miRNA |
| --- | --- |
| CD59 | hsa-miR-17-5p |
| CD59 | hsa-miR-18a-5p |
| CD59 | hsa-miR-19a-3p |
| CD59 | hsa-miR-19b-3p |
| CD59 | hsa-miR-20a-5p |
| CD59 | hsa-miR-25-3p |
| CD59 | hsa-miR-33a-5p |
| CD59 | hsa-miR-92a-3p |
| CD59 | hsa-miR-93-5p |
| CD59 | hsa-miR-103a-3p |
| CD59 | hsa-miR-106a-5p |
| CD59 | hsa-miR-107 |
| CD59 | hsa-miR-192-5p |
| CD59 | hsa-miR-183-5p |
| CD59 | hsa-miR-186-5p |
| CD59 | hsa-miR-194-5p |
| CD59 | hsa-miR-106b-5p |
| CD59 | hsa-miR-324-3p |
| CD59 | hsa-miR-345-5p |
| CD59 | hsa-miR-576-5p |
| CD59 | hsa-miR-590-5p |
| CD59 | hsa-miR-425-5p |
| CD59 | hsa-miR-769-5p |
| CD59 | hsa-miR-423-5p |
| CD59 | hsa-miR-556-3p |
| CD59 | hsa-miR-589-5p |
| CD59 | hsa-miR-942-5p |
| HLA-B | hsa-miR-128-3p |
| HLA-B | hsa-miR-130b-3p |
| HLA-B | hsa-miR-1306-5p |
| NDST1 | hsa-miR-96-5p |
| NDST1 | hsa-miR-29b-3p |
| NDST1 | hsa-miR-29c-3p |
| NDST1 | hsa-miR-148b-3p |
| NDST1 | hsa-miR-625-5p |
| PLXNB2 | hsa-miR-7-5p |
