# Supplementary material for: Gene signatures associated with exosomes as diagnostic markers of postpartum depression and their role in immune infiltration
Source: Front Endocrinol (Lausanne). 2025 Jul 17;16:1542327. doi: 10.3389/fendo.2025.1542327 (PMC12310459; doi:10.3389/fendo.2025.1542327)
Supplement: Supplementary file 7 [file Table7.docx]

### Table 7. mRNA-RBP interaction network nodes.

| mRNA | RBP |
| --- | --- |
| HNRNPA2B1 | CSTF2 |
| HNRNPA2B1 | ELAVL1 |
| HNRNPA2B1 | FIP1L1 |
| HNRNPA2B1 | FUS |
| HNRNPA2B1 | HNRNPA2B1 |
| HNRNPA2B1 | HNRNPC |
| HNRNPA2B1 | KHDRBS2 |
| HNRNPA2B1 | NUDT21 |
| HNRNPA2B1 | PTBP1 |
| HNRNPA2B1 | RBM10 |
| HNRNPA2B1 | RBMX |
| HNRNPA2B1 | RNPS1 |
| HNRNPA2B1 | SCAF4 |
| HNRNPA2B1 | SCAF8 |
| HNRNPA2B1 | U2AF1 |
| HNRNPA2B1 | U2AF2 |
| HNRNPA2B1 | YTHDF1 |
| NDST1 | CSTF2T |
| NDST1 | CTCF |
| NDST1 | ELAVL1 |
| NDST1 | FAM120A |
| NDST1 | HNRNPA2B1 |
| NDST1 | HNRNPC |
| NDST1 | IGF2BP2 |
| NDST1 | RBFOX2 |
| NDST1 | RBMX |
| PLXNB2 | ALYREF |
| PLXNB2 | DDX54 |
| PLXNB2 | MTDH |
| PLXNB2 | PRPF8 |
| PLXNB2 | RBFOX2 |
| PLXNB2 | RNPS1 |
| PLXNB2 | SCAF4 |
| PLXNB2 | SCAF8 |
| SCARB1 | CSTF2T |
| SCARB1 | ELAVL1 |
| SCARB1 | FAM120A |
| SCARB1 | HNRNPC |
| SCARB1 | HNRNPK |
| SCARB1 | IGF2BP2 |
| SCARB1 | NONO |
| SCARB1 | PTBP1 |
| SCARB1 | RBFOX2 |
| SCARB1 | RBMX |
| SCARB1 | SCAF8 |
| SCARB1 | TARDBP |
| TPP2 | ALYREF |
| TPP2 | CSTF2 |
| TPP2 | CSTF2T |
| TPP2 | ELAVL1 |
| TPP2 | HNRNPC |
| TPP2 | IGF2BP2 |
| TPP2 | RBMX |
| TPP2 | RNPS1 |
| TPP2 | SCAF8 |
| TPP2 | TARDBP |
| TPP2 | U2AF1 |
| TPP2 | U2AF2 |

RBP，RNA binding protein。
