# Supplementary material for: Gene signatures associated with exosomes as diagnostic markers of postpartum depression and their role in immune infiltration
Source: Front Endocrinol (Lausanne). 2025 Jul 17;16:1542327. doi: 10.3389/fendo.2025.1542327 (PMC12310459; doi:10.3389/fendo.2025.1542327)
Supplement: Supplementary file 8 [file Table8.docx]

### Table 8. mRNA-TF interaction network nodes.

| mRNA | TF |
| --- | --- |
| TPP2 | FOS |
| TPP2 | FOSL2 |
| TPP2 | FOXA1 |
| TPP2 | JUN |
| TPP2 | JUND |
| TPP2 | NR3C1 |
| TPP2 | RAD21 |
| TPP2 | RUNX1 |
| TPP2 | SPI1 |
| TPP2 | CEBPB |
| TPP2 | AR |
| TPP2 | CTCF |
| AKR1B1 | FOS |
| AKR1B1 | FOSL2 |
| AKR1B1 | FOXA1 |
| AKR1B1 | JUN |
| AKR1B1 | JUND |
| AKR1B1 | NR3C1 |
| AKR1B1 | RAD21 |
| AKR1B1 | RUNX1 |
| AKR1B1 | SPI1 |
| AKR1B1 | CEBPB |
| AKR1B1 | AR |
| AKR1B1 | CTCF |
| CD59 | CTCF |
| CD59 | FOXA1 |
| CD59 | GATA1 |
| CD59 | RAD21 |
| CD59 | SPI1 |
| CD59 | TAL1 |
| FAH | ELF1 |
| HNRNPA2B1 | CTCF |
| HNRNPA2B1 | EGR1 |
| HNRNPA2B1 | MYC |
| HNRNPA2B1 | POLR2A |
| HNRNPA2B1 | SPI1 |
| HNRNPA2B1 | YY1 |
| HNRNPA2B1 | CEBPB |
| HNRNPA2B1 | CREB1 |
| NDST1 | JUN |
| NDST1 | JUND |
| NDST1 | NR3C1 |
| NDST1 | EP300 |
| NDST1 | FOS |
| NDST1 | FOSL2 |
| PARK7 | CREB1 |
| PARK7 | ERG |
| PARK7 | MAX |
| PARK7 | NRF1 |
| PARK7 | RELA |
| PLXNB2 | ELF1 |
| PLXNB2 | ERG |
| PLXNB2 | FOS |
| PLXNB2 | FOSL2 |
| PLXNB2 | FOXA1 |
| PLXNB2 | FOXA2 |
| PLXNB2 | GABPA |
| PLXNB2 | JUN |
| PLXNB2 | JUND |
| SCARB1 | CEBPB |
| SCARB1 | ESR1 |
| SCARB1 | FOS |
| SCARB1 | FOXA1 |
| SCARB1 | FOXA2 |
| SCARB1 | GATA1 |
| SCARB1 | HNF4A |
| SCARB1 | MAX |
| SCARB1 | MYC |
| SCARB1 | SPI1 |
| SCARB1 | TAL1 |
| TPP2 | ELF1 |
| TPP2 | FOXA2 |
| TPP2 | GABPA |
| TPP2 | USF1 |
| TPP2 | USF2 |

TF：Transcription factors。
